# Abyssal fauna of the UK-1 polymetallic nodule exploration area, Clarion-Clipperton Zone, central Pacific Ocean: Mollusca

**DOI:** 10.3897/zookeys.707.13042

**Published:** 2017-10-10

**Authors:** Helena Wiklund, John D. Taylor, Thomas G. Dahlgren, Christiane Todt, Chiho Ikebe, Muriel Rabone, Adrian G. Glover

**Affiliations:** 1 Life Sciences Department, Natural History Museum, London SW7 5BD, UK; 2 Uni Research, Bergen, Norway; 3 Department of Marine Sciences, University of Gothenburg, Box 463, 40530 Gothenburg, Sweden; 4 University Museum of Bergen, University of Bergen, Allégt. 41, 5007 Bergen, Norway; 5 Rådgivande Biologer AS, Bredsgården, Bryggen 5003 Bergen, Norway

**Keywords:** New species, Bivalvia, Caudofoveata, Monoplacophora, Polyplacophora, Scaphopoda, Solenogastres, Aplacophora

## Abstract

We present the first DNA taxonomy publication on abyssal Mollusca from the Clarion-Clipperton Zone (CCZ), central Pacific ocean, using material collected as part of the Abyssal Baseline (ABYSSLINE) environmental survey cruise ‘AB01’ to the UK Seabed Resources Ltd (UKSRL) polymetallic-nodule exploration area ‘UK-1’ in the eastern CCZ. This is the third paper in a series to provide regional taxonomic data for a region that is undergoing intense deep-sea mineral exploration for high-grade polymetallic nodules.

Taxonomic data are presented for 21 species from 42 records identified by a combination of morphological and genetic data, including molecular phylogenetic analyses. These included 3 heterodont bivalves, 5 protobranch bivalves, 4 pteriomorph bivalves, 1 caudofoveate, 1 monoplacophoran, 1 polyplacophoran, 4 scaphopods and 2 solenogastres. Gastropoda were recovered but will be the subject of a future study. Seven taxa matched published morphological descriptions for species with deep Pacific type localities, and our sequences provide the first genetic data for these taxa. One taxon morphologically matched a known cosmopolitan species but with a type locality in a different ocean basin and was assigned the open nomenclature ‘*cf*’ as a precautionary approach in taxon assignments to avoid over-estimating species ranges. One taxon is here described as a new species, *Ledella knudseni* sp. n. For the remaining 12 taxa, we have determined them to be potentially new species, for which we make the raw data, imagery and vouchers available for future taxonomic study. The Clarion-Clipperton Zone is a region undergoing intense exploration for potential deep-sea mineral extraction. We present these data to facilitate future taxonomic and environmental impact study by making both data and voucher materials available through curated and accessible biological collections.

## Introduction

The abyssal zone of the world’s oceans has been defined as that between 3000 m and 6000 m depth, a bathymetric zone that encompasses 54% of the geographic surface of the planet (Smith et al. 2008). Molluscs form a characteristic and abundant group in this region, and many of them, most prominently among the bivalves, are deposit feeders that can sustain themselves on the steady rain of organic matter from surface regions. Current online databases list 1204 mollusc species recorded at abyssal depths from between 3000 m and 6000 m (OBIS 2017) out of a total of 3229 accepted ‘deep-sea’ mollusc species recorded from depths greater than 500 m (Glover et al. 2017).

The Clarion-Clipperton Zone (hereafter, CCZ) is so called as it lies between the Clarion and Clipperton Fracture Zones, topographical highs that extend longitudinally across almost the entire Pacific Ocean. There is no strict definition of the region, but it has come to be regarded as the area between these fracture zones that lies within international waters and encompasses the main areas of commercial interest for polymetallic nodule mining. Exploration licenses issued by the International Seabed Authority (ISA 2017) extend from 115°W (the easternmost extent of the UK-1 exploration area) to approximately 158°W (the westernmost extent of the COMRA exploration area), as such we use from hereafter a working definition of the CCZ as the box: 13°N158°W; 18°N118°W; 10°N112°W; 2°N155°W. This is an area of almost exactly 5 million sq km, approximately 1.4% of the ocean’s surface.

The Challenger expedition between 1872 and 1876 is said to be the start of modern oceanography, and in total about 4700 new species were described from it. However, in the Pacific Ocean they went from Japan to the Hawaiian Islands and after that fairly straight south down to about 40°S where they turned towards Valparaiso in Chile, and thus they did only touch the western-most part of the CCZ (Tizard et al. 1885). From 1891 to 1905 Agassiz did three expeditions onboard Albatross, after which Dall described 218 new species of molluscs and brachiopods from off the coast of Central and South America (Dall 1908). The Danish Galathea II deep-sea expedition went around the world in 1950-1952, but in the Pacific they went from New Zealand to Hawaii and then up north towards San Fransisco (Bruun et al. 1956), and did not collect anything in the actual CCZ.

**Figure 1.**
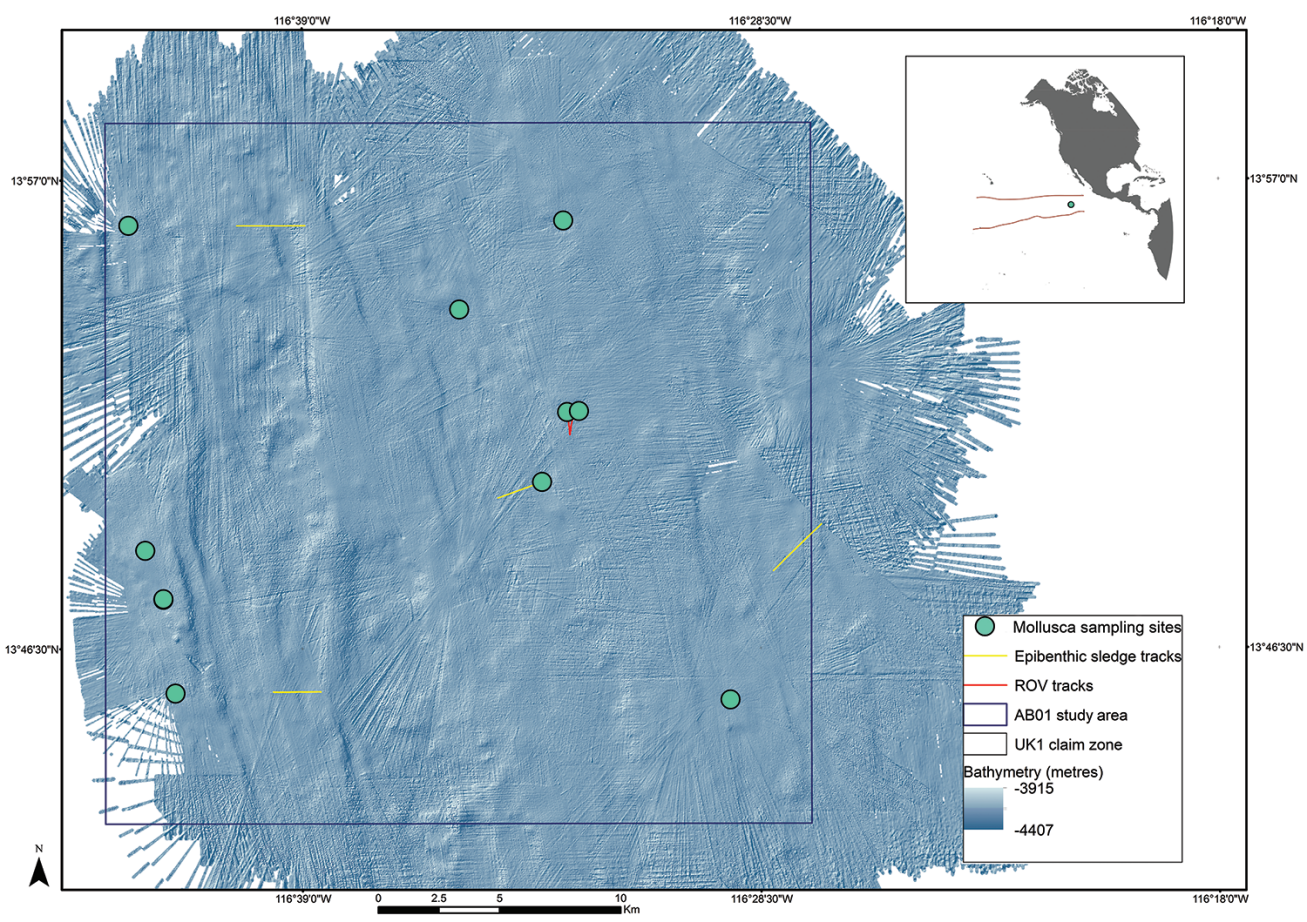
The UK Seabed Resources Ltd ‘UK-1’ polymetallic nodule exploration contract area ABYSSLINE (AB01) Stratum A, a 30 × 30 km survey box in the northern sector of the 58,000 km^2^ exploration area. Bathymetric survey and sample localities from the AB01 RV *Melville* survey cruise, October 2013, data courtesy Craig R. Smith (University of Hawaii), UK Seabed Resources Ltd and Seafloor Investigations, LLC.

Within the entire 5 million sq km CCZ, as defined above, online databased sources prior to this publication list only one benthic mollusc record when specifying depth between 3000-6000 m, and a further four records just south of CCZ (OBIS 2017). This result is due to lack of sampling and/or taxonomic knowledge given that an abundant and diverse mollusc fauna is suspected in the region based on anecdotal reports from past environmental surveys (e.g. ISA 1999; Ebbe et al. 2010). The goal of the DNA taxonomy part of the Abyssal Baseline (ABYSSLINE) program is to start to rectify these gaps in our knowledge and make data publically available that will eventually allow for a complete taxonomic synthesis of the CCZ supported by openly-available molecular and morphological data. We present results from a DNA taxonomy survey of abyssal benthic Mollusca collected as part of the first ABYSSLINE environmental survey cruise ‘AB01’ to the UK Seabed Resources Ltd (UKSRL) polymetallic nodule exploration contract area ‘UK-1’ (Fig. 1) in the eastern Clarion-Clipperton Zone (CCZ), central Pacific Ocean (Smith et al. 2013). Here we provide the first version of the Mollusca taxonomic synthesis, consisting of taxon records, images, genetic data and short descriptions from the first research cruise (AB01) aboard the RV *Melville* in October 2013. Gastropoda is not included in this version (subject to a future study), and we report on Bivalvia, Caudofoveata, Monoplacophora, Polyplacophora, Scaphopoda and Solenogastres.

This paper aims to provide regional taxonomic information for an area that is undergoing intense deep-sea mineral exploration for high-grade polymetallic nodules regulated by Sponsoring States (here the United Kingdom Government) and the International Seabed Authority (ISA 2017). The study is not a comprehensive faunal guide to the region, but a taxonomic data paper that will be updated with new additions following future collections and analyses. This publication is supported by similar data publications on other taxa from the CCZ. Two have been published (Echinodermata, Glover et al. 2016b and Cnidaria, Dahlgren et al. 2016), while other taxa are in preparation, forming a suite of taxonomic syntheses of biodiversity in the region, supported by a contract between the company UK Seabed Resources Ltd and the Natural History Museum, London and Uni Research, Bergen.

## Materials and methods

Knowledge of baseline biodiversity and biogeography in the CCZ is severely hampered by a lack of modern DNA-supported taxonomic studies (Glover et al. 2016a). With this in mind, three fundamental principles underpin our methodological pipeline: (1) the careful sorting and collection of live samples at sea using a ‘cold-chain’ pipeline by trained taxonomists, (2) the use of combined multiple-marker DNA sequences and morphological data in phylogenetics-based species descriptions or re-descriptions/records and (3) integrated data and sample management to push openly-available taxonomic data through online repositories linked to curated molecular and morphological collections in national museums.

### Fieldwork

The ABYSSLINE environmental baseline survey consists of a series of 30 × 30 km survey boxes (strata), three within the UK-1 exploration area, and an additional reference site outside the exploration area (Smith et al. 2013). Within each survey box, sample sites for a variety of benthic sampling gears are selected randomly – a randomized, stratified sampling design that assumes no *a priori* knowledge of the benthic environment. The UK-1 strata are being sampled in a series of oceanographic cruises during the course of the project, which commenced in July 2013, with the first cruise (AB01) taking place in October 2013 aboard the RV *Melville* (hereafter, cruise ‘AB01’). During this cruise, the first stratum was comprehensively mapped and sampled for a range of environmental and geophysical parameters (Fig. 1, Smith et al. 2013).

A comprehensive description of our DNA taxonomy pipeline is provided in Glover et al. (2016a). In summary, deep-sea benthic specimens from the AB01 strata were collected using a range of oceanographic sampling gears including box core (BC), epibenthic sledge (EBS), remotely operated vehicle (ROV) and multiple core (MC). Geographic data from sampling activities was recorded on a central GIS database (Fig. 1). Live-sorting of specimen samples was carried out aboard the RV *Melville* in a ‘cold-chain’ pipeline, in which material was immediately transferred and maintained in chilled, filtered seawater held at 2-4°C. Specimens were preliminary identified at sea and imaged live using stereomicroscopes with attached digital cameras. The specimens were then transferred to individual microtube vials containing an aqueous solution of 80% non-denatured ethanol, numbered and barcoded into a database and kept chilled until return to the Natural History Museum, London.

### Laboratory work

In the laboratory, specimens were re-examined using stereo and compound microscopes, identified and described to best possible taxonomic level with key morphological features photographed with digital cameras and a small tissue-sample taken for DNA extraction.

Extraction of DNA was done with DNeasy Blood and Tissue Kit (Qiagen) using a Hamilton Microlab STAR Robotic Workstation. About 1800 bp of 18S, 450 bp of 16S, and 650 bp of cytochrome c oxidase subunit I (COI) were amplified using primers listed in Table 1. PCR mixtures contained 1 µl of each primer (10 µM), 2 µl template DNA and 21 µl of Red Taq DNA Polymerase 1.1X MasterMix (VWR) in a mixture of total 25 µl. The PCR amplification profile consisted of initial denaturation at 95°C for 5 min, 35 cycles of denaturation at 94°C for 45 s, annealing at 55°C for 45 s, extension at 72°C for 2 min, and a final extension at 72°C for 10 min. PCR products were purified using Millipore Multiscreen 96-well PCR Purification System, and sequencing was performed on an ABI 3730XL DNA Analyser (Applied Biosystems) at The Natural History Museum Sequencing Facility, using the same primers as in the PCR reactions plus two internal primers for 18S (Table 1). Overlapping sequence fragments were merged into consensus sequences using Geneious (Kearse et al. 2012) and aligned using MAFFT (Katoh et al. 2002) for 18S and 16S, and MUSCLE (Edgar 2004) for COI, both programs used as plugins in Geneious, with default settings. Bayesian phylogenetic analyses (BA) were conducted with MrBayes 3.2 (Ronquist et al. 2012). Analyses were run for 10-30 million generations, of which the first 25% generations were discarded as burn-in.

**Table 1.** Primers used for PCR and sequencing of 18S, COI and 16S.

Primer	Sequence 5’-3’	Reference
**18S**		
18SA	AYCTGGTTGATCCTGCCAGT	Medlin et al. 1988
18SB	ACCTTGTTACGACTTTTACTTCCTC	Nygren and Sundberg 2003
620F	TAAAGYTGYTGCAGTTAAA	Nygren and Sundberg 2003
1324R	CGGCCATGCACCACC	Cohen et al. 1998
**COI**		
LCO1490	GGTCAACAAATCATAAAGATATTGG	Folmer et al. 1994
HCO2198	TAAACTTCAGGGTGACCAAAAAATCA	Folmer et al. 1994
**16S**		
ann16SF	GCGGTATCCTGACCGTRCWAAGGTA	Sjölin et al. 2005
16SbrH	CCGGTCTGAACTCAGATCACGT	Palumbi et al. 1996

### Data handling

The field and laboratory work created a series of databases and sample sets that are integrated into a data-management pipeline. This includes the transfer and management of data and samples between a central collections database, a molecular collections database and external repositories (GenBank, WoRMS, OBIS, GBIF, ZooBank) through DarwinCore archive. This provides a robust data framework to support DNA taxonomy, in which openly-available data and voucher material is key to quality data standards. A further elaboration of the data pipeline is published in Glover et al. (2016a).

### Taxonomic assignments

All future studies of biogeographic and bathymetric ranges, gene-flow, extinction risks, natural history, reproductive ecology, functional ecology and geochemical interactions of CCZ species are dependent on accurate identifications faciliated by taxonomy. This taxonomy is dependent on a sound theoretical underpinning – a species concept - coupled with the availability of both raw data and voucher samples. Here we use a phylogenetic species concept *sensu* Donoghue (1985) with species determined by DNA-based phylogenetic analysis and the recognition of distinct monophyletic groups as species. For those taxa where the typical morphological data that allows determination of species are missing, we provide the lowest-level taxonomic name possible, but include determination with genetic data. All materials (vouchers including archived frozen tissue) and genetic data are accessible together with the morphological data presented in this paper. A full list of all taxa including Natural History Museum Accession Numbers, NHM Molecular Collection Facility (NHM-MCF) FreezerPro numbers and NCBI GenBank Accession numbers is provided in Table 2.

**Table 2.** Taxon treatments presented in this paper. Includes Class, DNA Taxonomy ID (a species-level identification based on combined DNA and morphological evidence), GUID (Global Unique Identifier link to data record on http://data.nhm.ac.uk), ABYSSLINE Record number, NHM Accession number, NHM Molecular Collection Facility (MCF) sample ID number (NHMUK_MCF#) and NCBI GenBank accession number (Genbank#) for successfully sequenced genetic markers.

Class, sub-class	DNA Taxonomy ID	GUID#	ABYSS LINE record#	NHMUK Acc#	NHMUK MCF#	Gen Bank#
Bivalvia, Heterodonta	*Myonera* sp. (NHM_186)	45033e06-fb54-49d5-b632-767e63c1cfd3	NHM_186	20170037	175138970	MF157481 MF157508
Bivalvia, Heterodonta	*Thyasira* sp. (NHM_180)	49b2f599-bda4-4177-932f-59effe8a3320	NHM_051	20170038	175139015	MF157468 MF157501
Bivalvia, Heterodonta	*Thyasira* sp. (NHM_180)	b84e470d-73bc-413b-88f9-3d702509a37a	NHM_180	20170039	175139013	MF157478
Bivalvia, Heterodonta	*Vesicomya galatheae*	c609ed0c-f881-44c9-a6a0-3e36f0934997	NHM_143	20170040	175139017	MF157474
Bivalvia, Heterodonta	*Vesicomya galatheae*	314ef160-7cfa-4705-b091-640c3e69ad1a	NHM_255	20170041	175138995	MF157460 MF157487 MF157509
Bivalvia, Heterodonta	*Vesicomya galatheae*	3add2560-71c1-4879-afb8-0a5ed1449c89	NHM_260	20170042	175138988	MF157488 MF157510
Bivalvia, Protobranchia	*Bathyspinula calcar*	3ab74908-1a5d-465f-890c-49373a44906c	NHM_181	20170043	175138994	MF157479 MF157507
Bivalvia, Protobranchia	*Bathyspinula calcar*	61f15e3c-f070-48a1-b484-780b37f7feb6	NHM_146	20170044	175138993	MF157475 MF157505
Bivalvia, Protobranchia	*Bathyspinula calcar*	c44da298-9b61-4d6d-a1cd-2d6c3bd70859	NHM_149A	20170045	175138969	MF157506
Bivalvia, Protobranchia	*Bathyspinula calcar*	ad2cb87b-1fce-415d-ab45-1619bbc4352b	NHM_284	20170046	175139011	MF157514
Bivalvia, Protobranchia	*Ledella knudseni* sp. n.	8aec47f4-dcec-4668-8398-9e4b0c28ecb8	NHM_288A	20170047	175138963	MF157515
Bivalvia, Protobranchia	*Ledella knudseni* sp. n.	f1886d78-22bf-403e-bdb2-784b91c0eb12	NHM_288C	20170048	175139136	MF157491 MF157516
Bivalvia, Protobranchia	*Ledella* sp. (NHM_381)	8f077dac-baac-4fef-b6a1-7fd02d5f0070	NHM_381	20170049	175139009	MF157494 MF157521
Bivalvia, Protobranchia	*Ledella* sp. (NHM_381)	08d5c39f-b1e4-43d7-a8ea-2fe9abc05752	NHM_144	20170050	175139014	MF157458 MF157504
Bivalvia, Protobranchia	*Nucula profundorum*	f2133256-1cad-4255-a5cb-bd5331417127	NHM_141	20170051	175139038	MF157457 MF157473 MF157503
Bivalvia, Protobranchia	*Nucula profundorum*	f96a470e-237e-46b4-ba85-4c6196106071	NHM_274A	20170052	175138964	MF157512
Bivalvia, Protobranchia	*Nucula profundorum*	65f8d1ed-dd6a-4265-90d2-daf07491cd76	NHM_378	20170053	175138949	MF157464 MF157520
Bivalvia, Protobranchia	*Yoldiella* sp. (NHM_190)	621deeed-8f8a-4d2e-9136-4e30794fc68e	NHM_042	20170054	175139016	MF157467
Bivalvia, Protobranchia	*Yoldiella* sp. (NHM_190)	6dfa8946-aa7a-448d-9f4f-703a3b2a10d9	NHM_185	20170055	175138989	MF157480
Bivalvia, Protobranchia	*Yoldiella* sp. (NHM_190)	b6e48ff4-2e02-42dc-b9ed-286d297d1459	NHM_190	20170056	175138965	MF157482
Bivalvia, Protobranchia	*Yoldiella* sp. (NHM_190)	7a6c76df-989b-4fcd-9e9c-a442d0a02443	NHM_194	20170057	175139019	MF157485
Bivalvia, Protobranchia	*Yoldiella* sp. (NHM_190)	37b2493a-a725-4ec4-a720-cc9dd12fb49d	NHM_246	20170058	175139012	MF157486
Bivalvia, Protobranchia	*Yoldiella* sp. (NHM_190)	17d54bb4-9f38-4073-9bb6-17637773b058	NHM_289	20170059	175139034	MF157492 MF157517
Bivalvia, Protobranchia	*Yoldiella* sp. (NHM_190)	8923576e-4542-4fc7-9a89-016e8fb564cb	NHM_193	20170060	175139036	MF157484
Bivalvia, Pteriomorpha	Bentharca cf. asperula	9d29d7ec-55cd-4b41-929a-2379be221263	NHM_108	20170061	175138968	MF157470 MF157502
Bivalvia, Pteriomorpha	Bentharca cf. asperula	96bfe548-f511-49c4-b2a3-0a9a45f9154b	NHM_150	20170062	175138966	MF157476
Bivalvia, Pteriomorpha	Bentharca cf. asperula	8d9beefd-2fbc-4204-9bf8-90551419ac1c	NHM_170	20170063	175139018	MF157477
Bivalvia, Pteriomorpha	Bentharca cf. asperula	ccdd114d-c8a8-47da-ba84-8b8ca5125a6a	NHM_282	20170064	175139035	MF157490 MF157513
Bivalvia, Pteriomorpha	Bentharca cf. asperula	1d462c2a-bb98-4369-afc3-63a7c33a4bdd	NHM_427	20170065	175139023	MF157496
Bivalvia, Pteriomorpha	Bentharca cf. asperula	a30eab51-5f52-4fec-89d7-d47152895c92	NHM_454	20170066	175138984	MF157499
Bivalvia, Pteriomorpha	*Dacrydium panamensis*	180e485f-f1c2-41e1-b858-f02ba537804b	NHM_117	20170067	175138967	MF157471
Bivalvia, Pteriomorpha	*Limopsis* sp. (NHM_453)	ce9cbed0-82cc-420d-baad-fdfff7cc0986	NHM_453	20170069	175138999	MF157498 MF157524
Bivalvia, Pteriomorpha	*Catillopecten* sp. (NHM_105)	24f5c5bb-e419-48ef-baaa-4a6493f691d9	NHM_105	20170070	175138991	MF157469
Caudofoveata	Prochaetodermatidae sp. (NHM_344)	e68608f9-4b83-4eb9-89f2-0de4f89c21b0	NHM_344	20170071	175138997	MF157462
Monoplaco-phora	*Veleropilina oligotropha*	bf968b01-1991-43b7-87e4-25da4d5a9dc5	NHM_405	20170072	175138950	MF157465 MF157495 MF157522
Polyplaco-phora	*Leptochiton macleani*	d69b581d-8a79-4c4d-8f70-88b2ec07d86e	NHM_446	20170073	175139008	MF157466 MF157497 MF157523
Scaphopoda	*Fissidentalium* sp. (NHM_261)	679fa0ca-d647-446d-87c5-e8d33949efe2	NHM_261	20170074	175138971	MF157461 MF157489 MF157511
Scaphopoda	Gadilida sp. (NHM_192)	fc0e3ae8-9cce-46a0-bb8b-fafe0e2cb46b	NHM_192	20170075	175138946	MF157459 MF157483
Scaphopoda	*Gadila* sp. (NHM_345)	c301a72f-54cb-435e-8aae-17cf4d37675f	NHM_345	20170076	175138986	MF157463 MF157493 MF157518
Scaphopoda	Gadilida sp. (NHM_132)	6a1906d9-9ed1-4f6e-a0cf-2d53e2289a01	NHM_132	20170077	175138944	MF157456 MF157472
Solenogastres	Acanthomeniidae sp. (NHM_367)	c0577fc9-7302-4fec-bc8c-87a17a38bc91	NHM_367	20170078	175138973	MF157519
Solenogastres	Lophomeniinae sp. (NHM_027)	319fd186-b07f-4be7-986c-b96c20f63723	NHM_027	20170079	175139039	MF157500

## Systematics

### Bivalvia

#### Heterodonta

##### Anomalodesmata

###### Cuspidariidae Dall, 1886

####### *Myonera* Dall & E.A Smith, 1886

######## Myonera

Taxon classificationAnimaliaPholadomyoidaCuspidariidae

sp. (NHM_186)

######### Materials examined.

NHM_186 NHMUK 20170037, collected 2013-10-13, 13.93482 -116.55018, 4082 m. http://data.nhm.ac.uk/object/45033e06-fb54-49d5-b632-767e63c1cfd3

######### Description.

**Figure 2.**
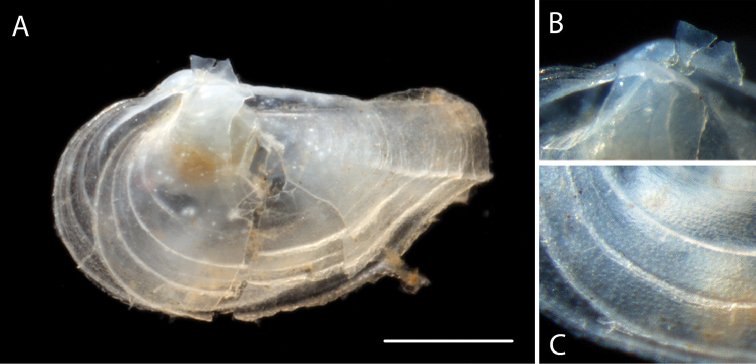
*Myonera* sp. (NHM_186) **A** Live specimen imaged at sea, slightly broken shell with live animal **B** Detail of hinge **C** Detail of shell ornamentation. Scale bar: 0.5 mm (**A**). Image attribution Glover, Dahlgren and Wiklund, 2017.

Shell thin, translucent, sub-ovate tapering posteriorly. Postero-dorsal margin straight. Rostrum short, demarcated by single, carinate radial rib. Sculpture of a few strong, widely spaced, commarginal lamellae, reduced on rostrum. Shell surface minutely pustulose (Fig. 2). Maximum length 1.5 mm, maximum height 1 mm.

######### Genetic data.

GenBank NHM_186 18S-MF157481, COI-MF157508.

######### Remarks.

The species resembles the supposedly cosmopolitan form *Myonera alleni* Poutiers & Bernard, 1995, previously as *Myonera atlantica* (Allen & Morgan, 1981). However, the type locality for this species is from the deep north Atlantic and no genetic data are available for comparison. No similar species is recorded from deep water of the eastern Pacific. Forms a unique monophyletic clade with two other cuspidariid species distinct from all other AB01 specimens (Fig. 5). No genetic matches on GenBank.

######### Ecology.

Found in polymetallic nodule province.

##### Lucinida

###### Thyasiridae Dall, 1900

####### Thyasira Lamarck, 1818

######## Thyasira

Taxon classificationAnimaliaPholadomyoidaThyasiridae

sp. (NHM_180)

######### Material examined.

NHM_051 NHMUK 20170038, collected 2013-10-09, 13.8372 -116.55843, 4336 m. http://data.nhm.ac.uk/object/49b2f599-bda4-4177-932f-59effe8a3320

NHM_180 NHMUK 20170039, collected 2013-10-13, 13.93482 -116.55018, 4082 m. http://data.nhm.ac.uk/object/b84e470d-73bc-413b-88f9-3d702509a37a

######### Description.

**Figure 3.**
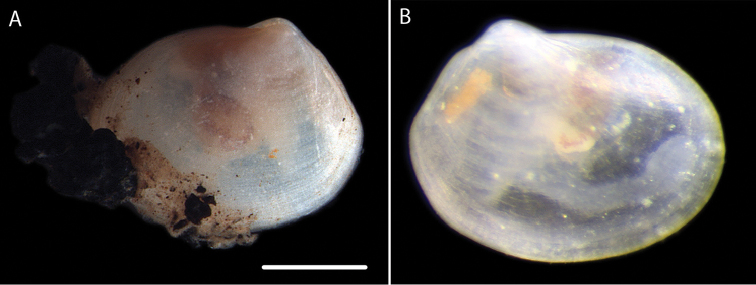
*Thyasira* sp. (NHM_180) **A** Preserved specimen (NHM_180) with pieces of polymetallic nodule adhered to shell margin **B** Additional small specimen (live imaged at sea) NHM_051. Scale bar: 0.5 mm (**A**). Image attribution Glover, Dahlgren and Wiklund, 2017.

Minute, thin-shelled, translucent, anteriorly extended, longer than high, umbones posterior of mid-line, posteriorly angulate, antero-dorsal margin long, evenly curved, shell surface smooth. Gill with single demibranch of about 10 widely spaced filaments, ventral edge of the gill does not cover the body pouches. Foot relatively large with distal bulb (Fig. 3). NHM_180 length 1.1 mm.

######### Genetic data.

GenBank NHM_051 18S-MF157468, COI-MF157501; NHM_180 18S-MF157478.

######### Remarks.

Forms a monophyletic clade with four other thyasirid species (Fig. 5) and distinct from all other AB01 specimens. No genetic matches on GenBank. Morphologically the species is similar in shape to abyssal thyasirid species (*Thyasira inflata*, *T. transversa*) from the south Atlantic described and placed in Thyasira (Mendicula) by Payne & Allen (1991) but not similar to the type species of Mendicula (Lucina) induta Hedley, 1907 = *M. memorata* Iredale, 1924) or the widespread *Mendicula ferruginosa* (Forbes, 1844). No similarly shaped species has been recorded from the abyssal eastern Pacific.

######### Ecology.

Found in polymetallic nodule province.

##### Veneroida

###### Vesicomyidae Dall & Simpson, 1901

####### *Vesicomya* Dall, 1886

######## Vesicomya galatheae

Taxon classificationAnimaliaPholadomyoidaVesicomyidae

(Knudsen, 1970)

######### Material examined.

NHM_143 NHMUK 20170040, collected 2013-10-11, 13.75833 -116.69852, 4080 m. http://data.nhm.ac.uk/object/c609ed0c-f881-44c9-a6a0-3e36f0934997

NHM_255 NHMUK 20170041, collected 2013-10-17, 13.75583 -116.48667, 4076 m. http://data.nhm.ac.uk/object/314ef160-7cfa-4705-b091-640c3e69ad1a

NHM_260 NHMUK 20170042.1-2, collected 2013-10-17, 13.75583 -116.48667, 4076 m. http://data.nhm.ac.uk/object/3add2560-71c1-4879-afb8-0a5ed1449c89

######### Description.

**Figure 4.**
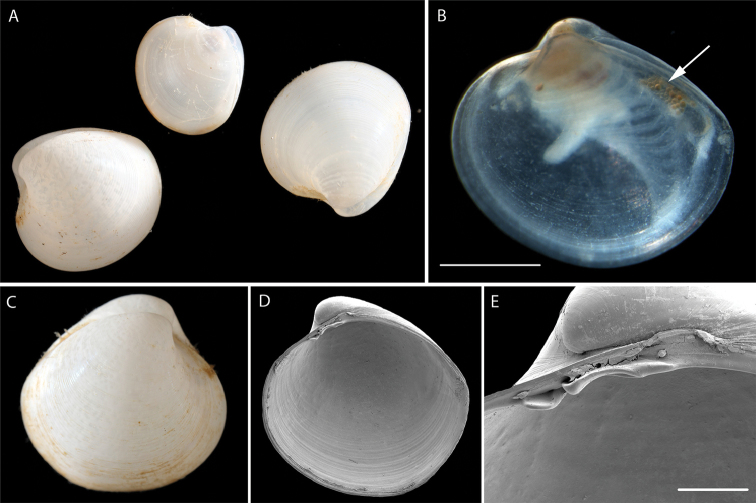
*Vesicomya galatheae* (Knudsen, 1970) **A** Live imaged specimens of NHM_260a,b,c habitus **B** Detail of NHM_143, probable juvenile, oil droplets arrowed **C** NHM_255 live imaged specimen **D–E** SEM detail of shell interior and hinge teeth of NHM_260a (right valve). Scale bars: 0.5 mm (**B, E**). Image attribution Glover, Taylor, Dahlgren & Wiklund, 2017.

Small, inflated sub-spherical. Sculpture of fine closely spaced low commarginal lamellae. Right valve with two cardinal teeth, posterior long, thin, anterior tooth small and short (Fig. 4). Specimen NHM_143 length 1.4 mm, height 1.2 mm.

######### Genetic data.

GenBank NHM_143 18S-MF157474; NHM_255 16S-MF157460, 18S-MF157487, COI-MF157509; NHM_260 18S-MF157488, COI-MF157510.

######### Remarks.

**Figure 5.**
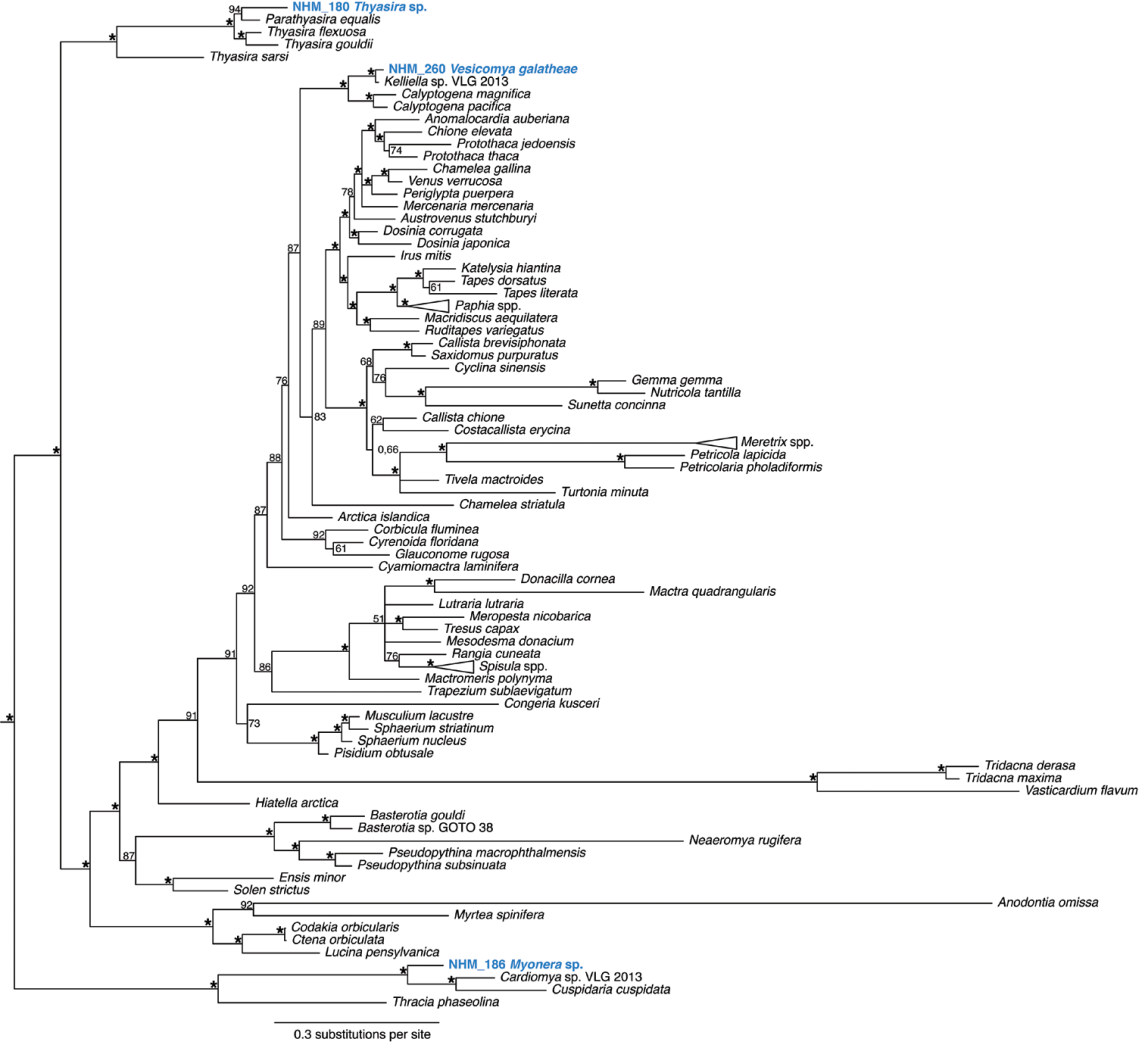
Phylogenetic analysis of Bivalvia: Heterodonta. 50% majority rule consensus tree from the Bayesian analyses using 18S and COI. Asterisks denotes support values of 95 or above.

*Vesicomya galatheae* was described from off Costa Rica and Panama at 2950- 3570 m. Morphologically similar to *Vesicomya pacifica* (Smith, 1885) holotype NHMUK 1887.2.9.2710-11 but Krylova et al. (2015) regard this as a northern Pacific species distinguished from *V. galatheae* by the shape, hinge teeth and number of siphonal tentacles. When comparing sequences from our CCZ specimens with the *Vesicomya pacifica* from Krylova et al. (2015), the K2P difference is 0.11. In the molecular tree (Fig. 5) it groups with a *Kelliella* species from the northwestern Atlantic and these two species form a sister clade to *Calyptogena* species. *Kelliella* species are very similar to *Vesicomya* and the relationships of species assigned to the two genera need clarification. Forms a unique monophyletic clade distinct from all other AB01 specimens. No genetic matches on GenBank.

######### Ecology.

Found in polymetallic nodule province.

#### Protobranchia

##### Nuculanoida

###### Bathyspinulidae Coan & Scott, 1997

####### *Bathyspinula* Allen & Sanders, 1982

######## Bathyspinula calcar

Taxon classificationAnimaliaPholadomyoidaBathyspinulidae

(Dall, 1908)

######### Material examined.

NHM_146 NHMUK 20170044, collected 2013-10-11, 13.75833 -116.69852, 4080 m. http://data.nhm.ac.uk/object/61f15e3c-f070-48a1-b484-780b37f7feb6

NHM_149A NHMUK 20170045, collected 2013-10-11, 13.75833 -116.69852, 4080 m. http://data.nhm.ac.uk/object/c44da298-9b61-4d6d-a1cd-2d6c3bd70859

NHM_181 NHMUK 20170043, collected 2013-10-13, 13.93482 -116.55018, 4082 m. http://data.nhm.ac.uk/object/3ab74908-1a5d-465f-890c-49373a44906c

NHM_284 NHMUK 20170046, collected 2013-10-17, 13.75583 -116.48667, 4076 m. http://data.nhm.ac.uk/object/ad2cb87b-1fce-415d-ab45-1619bbc4352b

######### Description.

**Figure 6.**
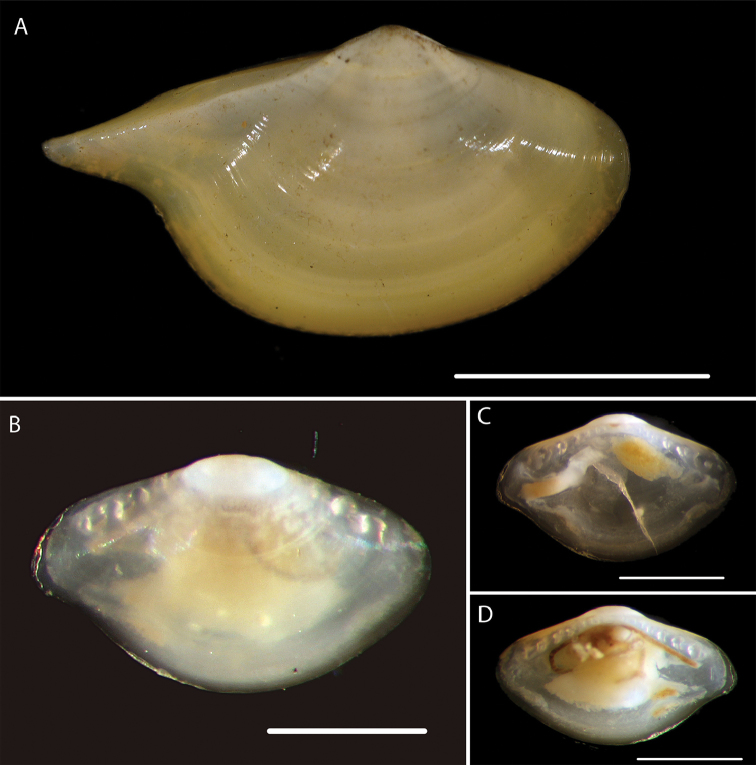
*Bathyspinula calcar* (Dall, 1908) **A** Specimen NHM_181, Image of live specimen after recovery, length 13.5 mm **B–D** Specimen NHM_149A confirmed juvenile *B. calcar* using DNA evidence, total length of animal ~2mm. Scale bars: 5 mm (**A**); 1 mm (**B–D**). Image attribution Glover, Taylor, Dahlgren & Wiklund, 2017.

Shell sub-ovate, laterally compressed, with long, sharply pointed posterior rostrum. Periostracum shiny, medium brown. Posterior rostrum shorter, less defined in juveniles. Voucher specimen NHM_181 shell length 13.5 mm, width 7.6 mm (Fig. 6A).

######### Genetic data.

GenBank NHM_146 18S-157475, COI-MF157505; NHM_149A COI-MF157506; NHM_181 18S-MF157479, COI-MF157507; NHM_284 COI-MF157514.

######### Remarks.

**Figure 7.**
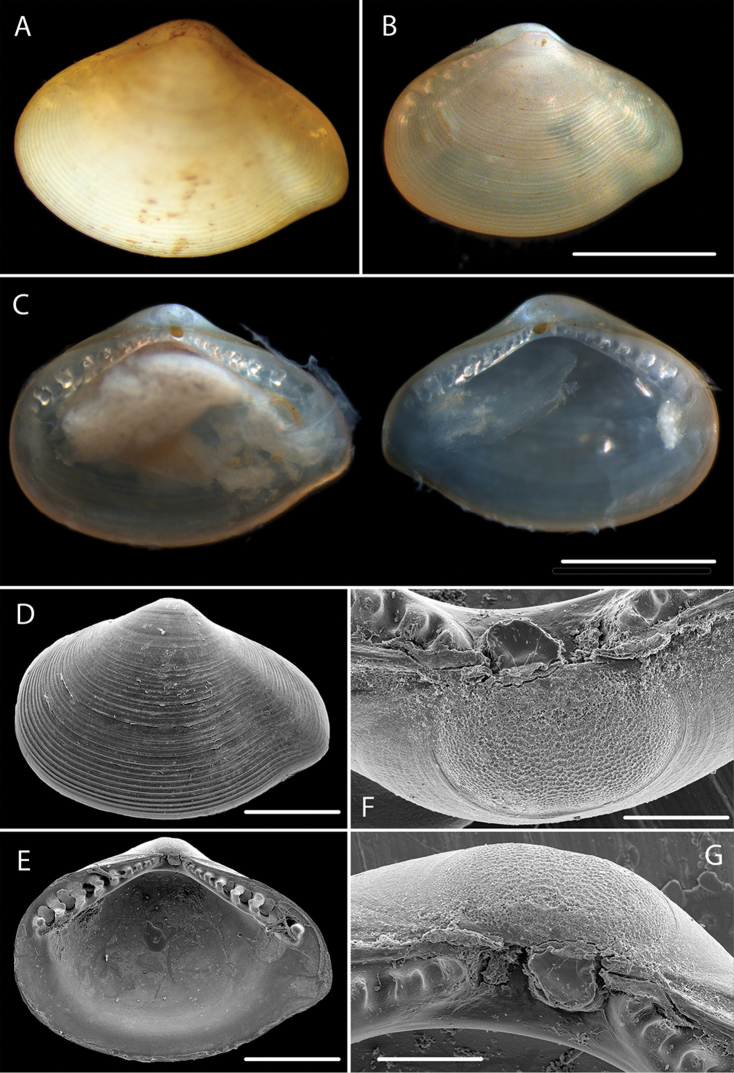
*Ledella knudseni* sp. n. **A** Holotype, specimen NHM_288c **B** Paratype, specimen NHM_288a **C** Specimen NHM 288a dissected prior to DNA sequencing and SEM **D–G** SEM of valve, hinge teeth and protoconch. Scale bars: 1 mm (**B–C**); 0.5 mm (**D–E**); 0.1 mm (**F–G**). Image attribution Glover, Taylor, Dahlgren & Wiklund, 2017.

Widely distributed in the eastern Pacific at depths of 400-5000 m (see Coan and Valentich-Scott 2012). The holotype (USNM 110573) was collected 725 km west of Trujillo, Peru at 2370 fathoms (4334 m). Forms a unique monophyletic clade distinct from all other AB01 specimens. Genetic match in 18S to *Bathyspinula calcar* (GenBank KC993875) from the north eastern Pacific (Sharma et al. 2013), but as the GenBank 18S sequence from *B. calcar* was only 289 bp long and as that specimen lacked COI, it was not included in the analyses. Some very small juvenile specimens (Fig. 6B–D) were recovered that superficially resemble *Ledella knudseni* sp. n. (Fig. 7) and may be easily confused. Genetic data confirmed these to be *Bathyspinula calcar* (Fig. 12). These may be distinguised from *Ledella* by the shiny and iridescent nature of the shell surface of *B. calcar*, which is preserved in the juveniles.

######### Ecology.

Relatively large bivalve recovered from epibenthic sledge tow in polymetallic nodule province.

##### Nuculanidae H. Adams & A. Adams, 1858

###### *Ledella* Verrill & Bush, 1897

####### Ledella knudseni

Taxon classificationAnimaliaPholadomyoidaNuculanidae

Taylor & Wiklund sp. n.

http://zoobank.org/66E692B5-7C61-4ADC-9539-EFC085424147

######## Material examined.

Paratype NHM_288A NHMUK 20170047.1-2, collected 2013-10-17, 13.75583 -116.48667, 4076 m. http://data.nhm.ac.uk/object/8aec47f4-dcec-4668-8398-9e4b0c28ecb8

Holotype NHM_288C NHMUK 20170048, collected 2013-10-17, 13.75583 -116.48667, 4076 m. http://data.nhm.ac.uk/object/f1886d78-22bf-403e-bdb2-784b91c0eb12

######## Description.

Shell relatively thick, robust. Ovoid with short rostrum, umbones broad, prominent; postero ventral margin sinuous; broad, shallow sulcus extending from umbones to posteroventral margin. Sculpture of low, relatively broad, closely spaced, commarginal lamellae; fine radial striations on rostrum and juvenile shell. Ligament internal, situated on broad resilium beneath umbones. Hinge robust, with 8-9 chevron shaped, blunt teeth to either side of ligament. Inner shell margin smooth. Prodissoconch large, ellipsoidal 0.3 mm long, with sharp rim, surface irregularly pitted. Holotype NHM_288C shell length 2.2 mm, width 1.5 mm; paratype NHM_288A shell length 2.1 mm, height 1.5 mm. (Figure 7).

######## Genetic data.

GenBank NHM_288A COI-MF157515; NHM_288C 18S-MF157491, COI-MF157516.

######## Remarks.

Similar in form to *Ledella ultima* (Smith, 1885) widespread in the abyssal Atlantic (Allen 2008), but has a less massive hinge with more teeth, 8-9 compared with 6-8 in *L. ultima*. Also similar is the species identified by Knudsen (1970) as *L. ultima* from the Sunda Trench in Indian Ocean at 3810 m. The only species recorded from the deep eastern Pacific is *Ledella dicella* (Dall, 1908) from 734-1200 m off Ecuador but this lacks the short rostrum and has 12-13 hinge teeth on each side of the ligament (Coan and Valentich-Scott 2012 pl. 26). No genetic matches on GenBank. *Ledella knudseni* groups in a small subclade with but is distinct from the Atlantic species *L. ultima* and *Ledella jamesi* Allen & Hannah, 1989, as well as another *Ledella* species from this study in the Pacific, *Ledella* sp. (NHM_381) (Figure 12). The new species can be confused with juveniles of *B. calcar* (see above), but shell is less shiny and iridescent, and ribs are more pronounced. DNA may be required to confirm identification.

######## Etymology.

Named for Jørgen Knudsen (1918-2009), deep-sea bivalve systematist and author of the Galathea Report on abyssal and hadal Bivalvia.

######## Ecology.

Found in polymetallic nodule province.

####### Ledella

Taxon classificationAnimaliaPholadomyoidaNuculanidae

sp. (NHM_381)

######## Material examined.

NHM_144 NHMUK 20170050, collected 2013-10-11, 13.75833 -116.69852, 4080 m. http://data.nhm.ac.uk/object/08d5c39f-b1e4-43d7-a8ea-2fe9abc05752

NHM_381 NHMUK 20170049, collected 2013-10-19, 13.93307 -116.71628, 4182 m. http://data.nhm.ac.uk/object/8f077dac-baac-4fef-b6a1-7fd02d5f0070

######## Description.

**Figure 8.**
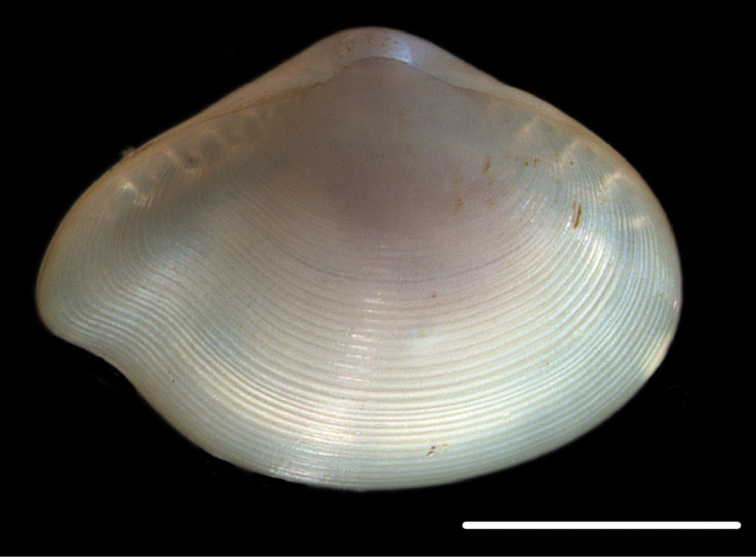
*Ledella* sp. (NHM_381). Scale bar: 1 mm. Image attribution Glover, Dahlgren & Wiklund, 2017.

Ovoid with short rostrum, shell shiny sub-translucent. Sculpture of fine closely spaced commarginal lamellae. Specimen NHM_381 length 2 mm (Fig. 8).

######## Genetic data.

GenBank NHM_144 16S-MF157458, COI-MF157504; NHM_381 18S-MF157494, COI-MF157521.

######## Remarks.

This species is morphologically very similar to the new *Ledella knudseni*, its sister taxon in the molecular phylogenetic analyses (Fig. 12), and DNA might be required to properly identify the species. No genetic matches on GenBank.

######## Ecology.

Found in polymetallic nodule province.

#### Nuculida

##### Nuculidae Gray, 1824

###### *Nucula* Lamarck, 1799

####### Nucula profundorum

Taxon classificationAnimaliaPholadomyoidaNuculidae

Smith, 1885

######## Material examined.

NHM_141 NHMUK 20170051, collected 2013-10-11, 13.75833 -116.69852, 4080 m. http://data.nhm.ac.uk/object/f2133256-1cad-4255-a5cb-bd5331417127

NHM_274A NHMUK 20170052, collected 2013-10-17, 13.75583 -116.48667, 4076 m. http://data.nhm.ac.uk/object/f96a470e-237e-46b4-ba85-4c6196106071

NHM_378 NHMUK 20170053.1-2, collected 2013-10-19, 13.93307 -116.71628, 4182 m. http://data.nhm.ac.uk/object/65f8d1ed-dd6a-4265-90d2-daf07491cd76

######## Description.

**Figure 9.**
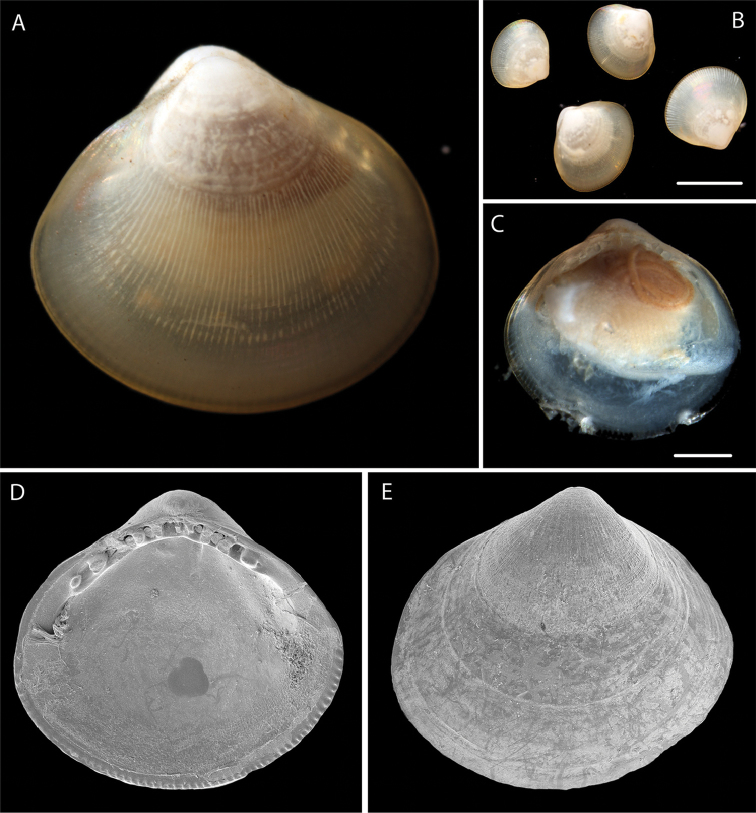
*Nucula profundorum* Smith, 1885 **A** Live specimen NHM_141 (for which 18S, CO1 and 16S sequences were obtained) **B** Live specimens NHM_274 (4 specimens from same sample) **C** Open shell from single individual NHM_274A with tissue sample taken for DNA sequencing **D–E** SEM of NHM_378 valve showing hinge teeth. Scale bars: 1.5 mm (**B**); 0.5 mm (**C**). Image attribution Glover, Taylor, Dahlgren & Wiklund, 2017.

Small, trigonal- subovate. Periostracum light brown, shiny. Sculpture of fine radial lirae. Resilifer small. Hinge teeth: 5 anterior, 4 posterior. Inner shell margin finely denticulate. Voucher NHM_274A width 2 mm, height 1.8 mm (Fig. 9).

######## Genetic data.

GenBank NHM_141 16S-MF157457, 18S-MF157473, COI-MF157503; NHM_274A COI-MF157512; NHM_378 16S-MF157464, COI-MF157520.

######## Remarks.

**Figure 10.**
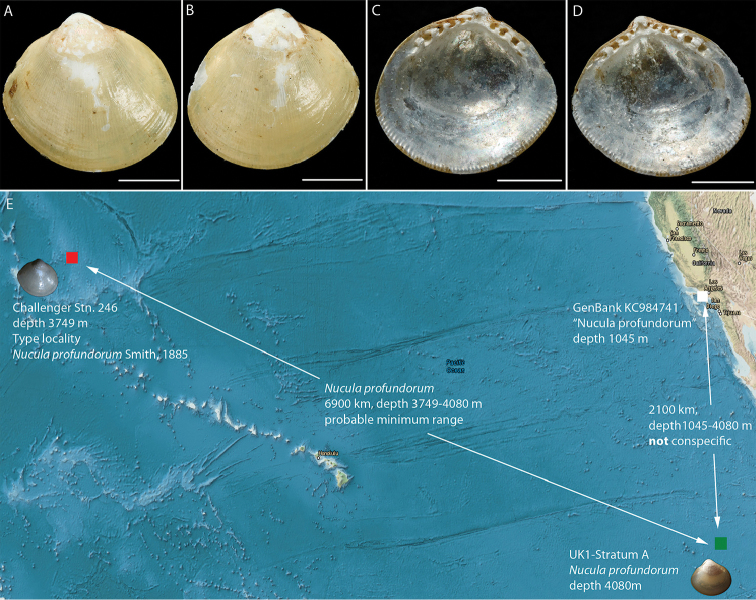
*Nucula profundorum* Smith, 1885. **A–D** Syntype BMNH 1887.2.9.2919, scalebars 1mm **E** Type locality (red) of *N. profundorum* from Challenger Expedition in relation to ABYSSLINE sampling location (green) and GenBank voucher specimen sampling location (white). Bathymetric data (**D**) from NOAA.

Morphologically matches *Nucula profundorum* Smith, 1885 based on examination of the syntype specimens [NHMUK 1887.2.9.2919]. In the molecular analysis of nuculoid protobranchs (Fig. 12) *Nucula profundorum* and the Atlantic *Nucula atacellana* Schenck, 1939 are well supported sister species. However the *N. profundorum* identified from the present samples differs genetically from the *N. profundorum* record in GenBank (accession nr KJ950274; Jennings and Etter 2014) which we believe may be misassigned. That sample came from 1045 m in the north eastern Pacific off San Diego (Figure 10). The shell illustrated by Coan and Valentich-Scott (2012 pl 12) as *N. profundorum* has more hinge teeth. There may be a complex of morphologically similar species in the eastern Pacific. No genetic matches on GenBank.

######## Ecology.

The most abundant bivalve mollusc recorded in the ABYSSLINE sampling programme, frequently found in epibenthic sledge and box core samples from region of sediment and polymetallic nodules.

##### Yoldiidae

###### *Yoldiella* A.E Verrill & Bush, 1897

####### Yoldiella

Taxon classificationAnimaliaPholadomyoidaYoldiidae

sp. (NHM_190)

######## Material examined.

NHM_042 NHMUK 20170054, collected 2013-10-09, 13.8372 -116.55843, 4336 m. http://data.nhm.ac.uk/object/621deeed-8f8a-4d2e-9136-4e30794fc68e

NHM_185 NHMUK 20170055, collected 2013-10-13, 13.93482 -116.55018, 4082 m. http://data.nhm.ac.uk/object/6dfa8946-aa7a-448d-9f4f-703a3b2a10d9

NHM_190 NHMUK 20170056, collected 2013-10-13, 13.93482 -116.55018, 4082 m. http://data.nhm.ac.uk/object/b6e48ff4-2e02-42dc-b9ed-286d297d1459

NHM_193 NHMUK 20170060, collected 2013-10-13, 13.93482 -116.55018, 4082 m. http://data.nhm.ac.uk/object/8923576e-4542-4fc7-9a89-016e8fb564cb

NHM_194 NHMUK 20170057, collected 2013-10-13, 13.93482 -116.55018, 4082 m. http://data.nhm.ac.uk/object/7a6c76df-989b-4fcd-9e9c-a442d0a02443

NHM_246 NHMUK 20170058, collected 2013-10-16, 13.81166 -116.71, 4076 m. http://data.nhm.ac.uk/object/37b2493a-a725-4ec4-a720-cc9dd12fb49d

NHM_289 NHMUK 20170059, collected 2013-10-17, 13.75583 -116.48667, 4076 m. http://data.nhm.ac.uk/object/17d54bb4-9f38-4073-9bb6-17637773b058

######## Description.

**Figure 11.**
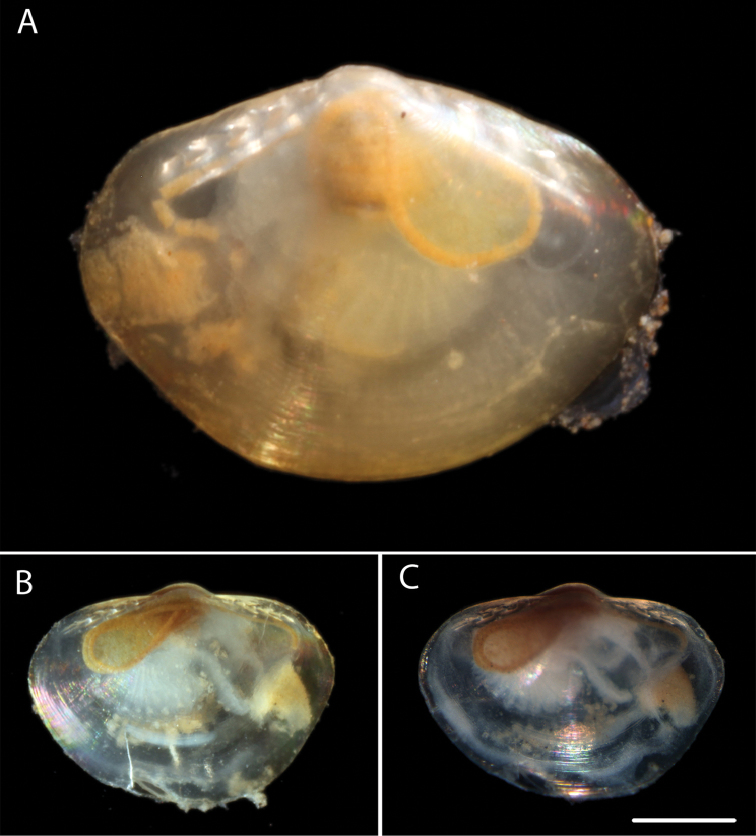
*Yoldiella* sp. (NHM_190) **A** Voucher specimen NHM_190 **B** Live specimens NHM_185 **C** NHM_185 after preservation in ethanol for 3 months prior to DNA sequencing. Scale bar: 0.5 mm (**C**). Image attribution Glover, Dahlgren & Wiklund, 2017.

Small, sub-ovate, longer than high, umbone at mid-line, dorsal margin horizontal to slightly curved, ventral margin deeply rounded, thin-shelled, shiny, semi-transparent, smooth except for growth increments. Internal features not investigated but 4-5 anterior and posterior chevron teeth. Hindgut visible though the shell forms a simple rounded loop on right side of body. DNA voucher NHM_190 shell length 1.6 mm, height 1 mm. Voucher specimen NHM_185 shell length 1.5 mm, height 1 mm (Fig. 11).

######## Genetic data.

GenBank NHM_042 18S-MF157467; NHM_185 18S-MF157480; NHM_190 18S-MF157482; NHM_193 18S-MF157484; NHM_194 18S-MF157485; NHM_246 18S-MF157486; NHM_289 18S-MF157492, COI-MF157517.

######## Remarks.

**Figure 12.**
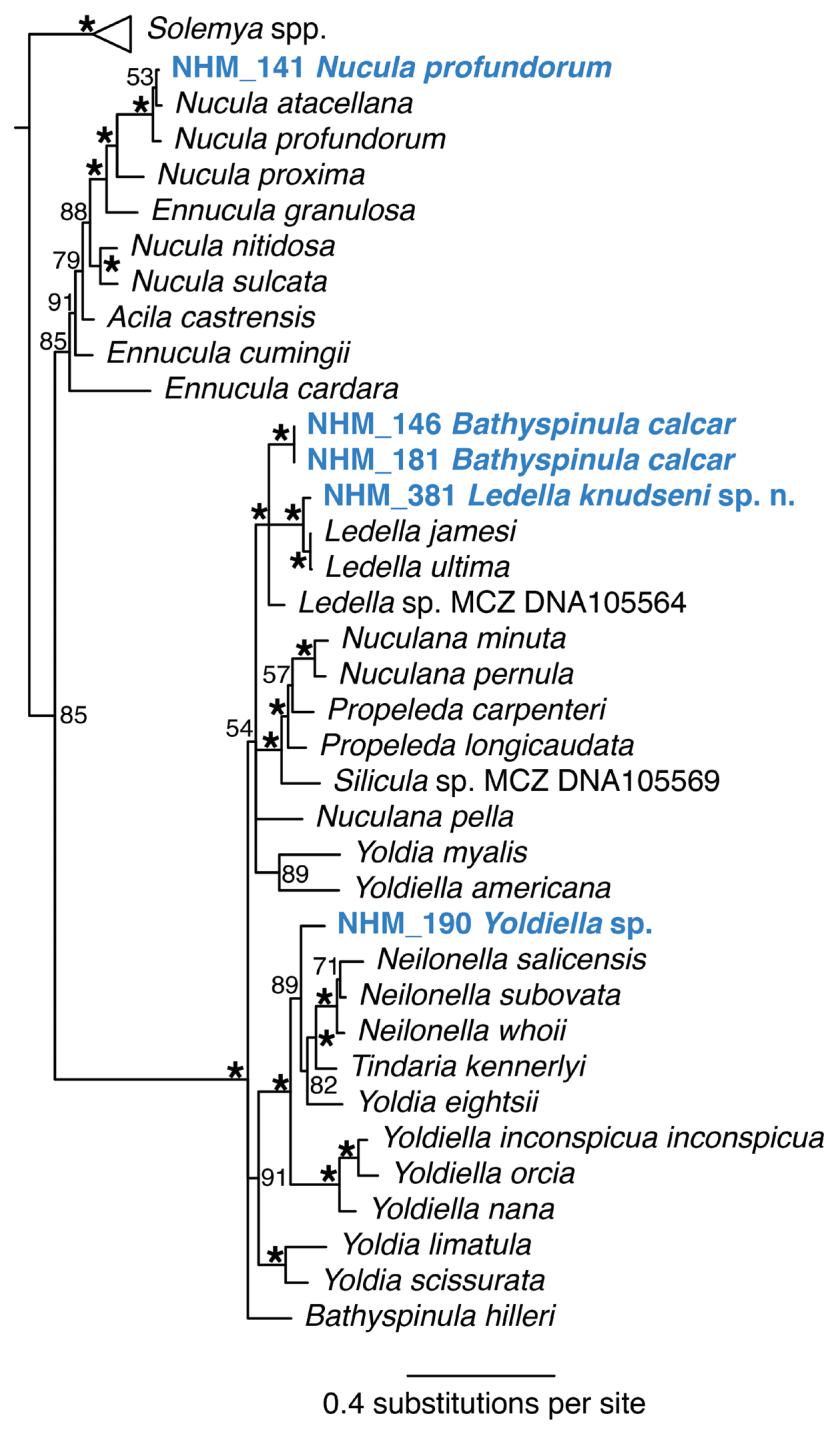
Phylogenetic analysis of Bivalvia: Protobranchia. 50% majority rule consensus tree from the Bayesian analyses using 18S and COI. Asterisks denotes support values of 95 or above.

Extremely small, semi-transparent bivalves typically about 1 mm in size. *Yoldiella* species are particularly difficult to identify (see Killeen and Turner 2009). Forms a unique monophyletic clade distinct from all other AB01 specimens. No genetic matches on GenBank. In the molecular tree (Fig. 12) the genus *Yoldiella* is not monophyletic, and the present species does not group with another Eastern Pacific bathyal species, *Yoldiella orcia* (Dall, 1916), which instead forms a well-supported subclade with two Atlantic species.

######## Ecology.

Found in polymetallic nodule province.

#### Pteriomorphia

##### Arcoida

###### Arcidae Lamarck, 1809

####### *Bentharca* Verrill & Bush, 1898

######## Bentharca cf. asperula

Taxon classificationAnimaliaPholadomyoidaArcidae

(Dall, 1881)

######### Material examined.

NHM_108 NHMUK 20170061, collected 2013-10-11, 13.79335 -116.70308, 4081 m. http://data.nhm.ac.uk/object/9d29d7ec-55cd-4b41-929a-2379be221263

NHM_150 NHMUK 20170062.1-2, collected 2013-10-11, 13.75833 -116.69852, 4080 m. http://data.nhm.ac.uk/object/96bfe548-f511-49c4-b2a3-0a9a45f9154b

NHM_170 NHMUK 20170063, collected 2013-10-11, 13.7936 -116.70308, 4078 m. http://data.nhm.ac.uk/object/8d9beefd-2fbc-4204-9bf8-90551419ac1c

NHM_282 NHMUK 20170064, collected 2013-10-17, 13.75583 -116.48667, 4076 m. http://data.nhm.ac.uk/object/ccdd114d-c8a8-47da-ba84-8b8ca5125a6a

NHM_427 NHMUK 20170065, collected 2013-10-20, 13.86367 -116.54432, 4050 m. http://data.nhm.ac.uk/object/1d462c2a-bb98-4369-afc3-63a7c33a4bdd

NHM_454 NHMUK 20170066, collected 2013-10-21, 13.90165 -116.59, 4163 m. http://data.nhm.ac.uk/object/a30eab51-5f52-4fec-89d7-d47152895c92

######### Description.

**Figure 13.**
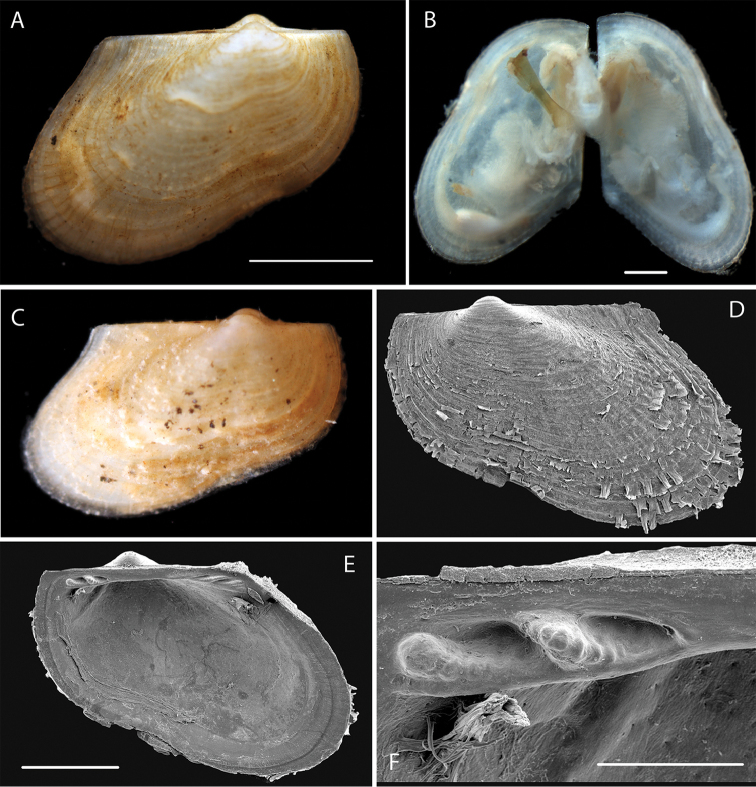
Bentharca cf. asperula (Dall, 1881) **A** Voucher specimen NHM_150 live after recovery **B** Specimen NHM_150 after preservation and dissection for DNA sample showing valves **C** Specimen NHM_108 Live **D–F** Specimen NHM_150 SEM showing shell ornamentation and hinge teeth. Scale bars: 1 mm (**A, E**); 0.5 mm (**B**); 0.2 mm (**F**). Image attribution Glover, Taylor, Dahlgren & Wiklund, 2017.

Shell elongate, trapezoidal, strongly inequilateral, anteriorly attentuated and posteriorly expanded, umbones small, low, dorsal edge straight. Byssal sinus in ventral margin. Sculpture of irregular commarginal lamellae and low radial ribs but covered by a thick, shaggy, brown periostracum with projecting scales. Two pre- and post- umbonal hinge teeth with each tooth crossed by transverse grooves giving a lobate appearance (Fig. 13E, F). Inner shell margin smooth. DNA voucher NHM_150 shell length 3.2 mm shell width 1.9 mm.

######### Genetic data.

GenBank NHM_108 18S-MF157470, COI-MF157502; NHM_150 18S-MF157476; NHM_170 18S-MF157477; NHM_282 18S-MF157490, COI-MF157513; NHM_427 18S-MF157496; NHM_454 18S-MF157499.

######### Remarks.

*Bentharca asperula* has been regarded as a cosmopolitan deep-water species with a considerable recorded depth range of 430–5005 m (Knudsen 1967, 1970, Coan and Valentich-Scott 2012) from Atlantic, Indian and Pacific Oceans. The lectotype and paralectotypes (USNM 63174, 887339, 94363) originated from the Gulf of Mexico, off Yucatan, 2868 m (Blake stn 33). Because of its epifaunal, byssate life habit *B. asperula* shows considerable shape variation and Knudsen (1967) synonymised several nominal species and described how the number of hinge teeth increases with shell size (age). Without supporting genetic evidence from samples from different oceans it is impossible to test whether the species is truly cosmopolitan. Perhaps significantly, no shell has been described with as few hinge teeth as the present sample and none with the transverse grooves (Fig. 13F). No genetic matches on GenBank.

######### Ecology.

Quite abundant. Found in polymetallic nodule province.

##### Mytiloida

###### Mytilidae Rafinesque, 1815

####### *Dacrydium* Torell, 1859

######## Dacrydium panamensis

Taxon classificationAnimaliaPholadomyoidaMytilidae

Knudsen, 1970

######### Material examined.

NHM_117 NHMUK 20170067, collected 2013-10-11, 13.79335 -116.70308, 4081 m. http://data.nhm.ac.uk/object/180e485f-f1c2-41e1-b858-f02ba537804b

######### Description.

**Figure 14.**
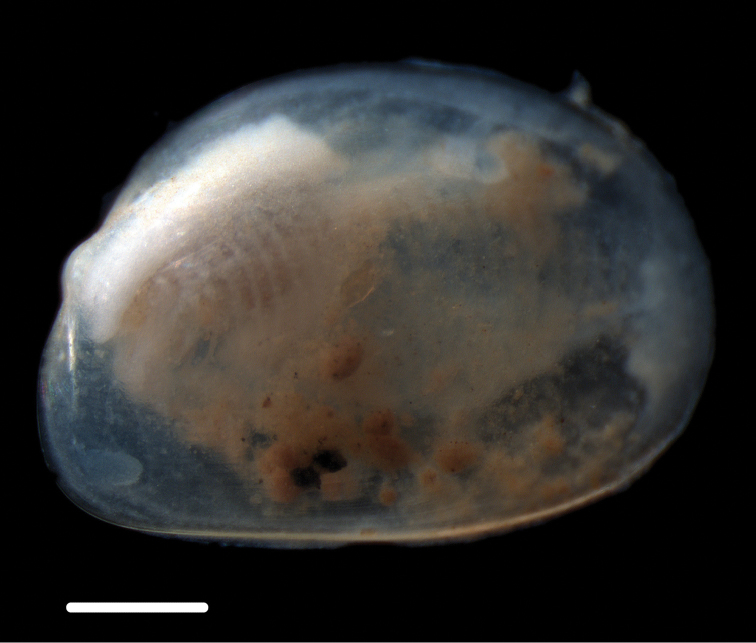
*Dacrydium panamensis* Knudsen, 1970 Specimen NHM_117. Scale bar: 0.5 mm. Image attribution Glover, Dahlgren & Wiklund, 2017.

Shell small, subovate, translucent, anterior-ventral margin slightly produced, highest point near mid-line. Voucher NHM_117 Shell length 1.7 mm, shell height 2.5 mm (Fig. 14).

######### Genetic data.

GenBank NHM_117 18S-MF157471.

######### Remarks.

Identified from figures in Knudsen (1970) and Coan & Valentich-Scott (2012). The holotype of *D. panamensis* was collected on the Galathea expedition (stn 726) at 3670-3270 m depth in Gulf of Panama. In the molecular analysis (Fig. 17) it aligns as a sister species to many shallow water Mytilidae. No genetic matches on GenBank.

######### Ecology.

Found in polymetallic nodule province.

###### Limopsidae Dall, 1895

####### *Limopsis* Sassi, 1827

######## Limopsis

Taxon classificationAnimaliaPholadomyoidaLimopsidae

sp. (NHM_453)

######### Material examined.

NHM_453 NHMUK 20170069.1-2, collected 2013-10-21, 13.90165 -116.59, 4163 m. http://data.nhm.ac.uk/object/ce9cbed0-82cc-420d-baad-fdfff7cc0986

######### Description.

**Figure 15.**
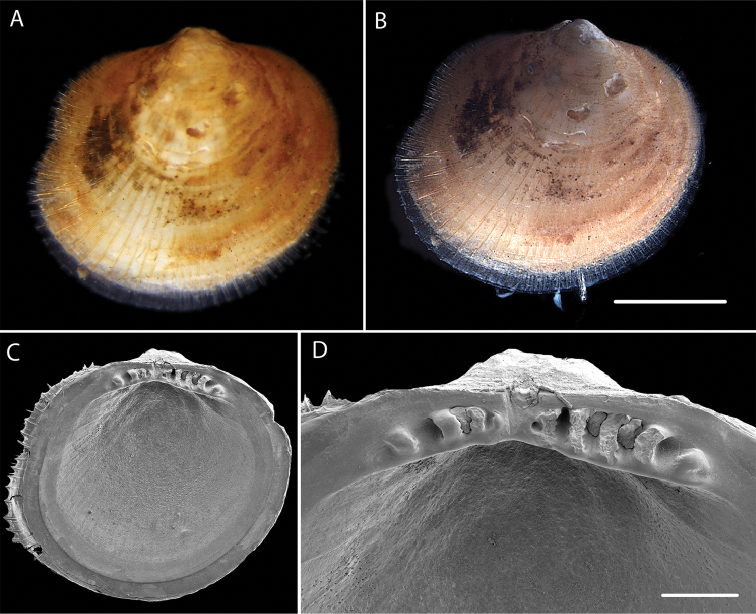
*Limopsis* sp. (NHM_453) **A** Specimen NHM_453 live after recovery **B** Specimen NHM_453 after preservation **C–D** SEM of interior of right valve showing hinge teeth. Scale bars: 2mm **B**, 0.5mm **D**. Image attribution Glover, Taylor, Dahlgren & Wiklund, 2017.

Subcircular to slightly oblique with slightly sinuous posterior margin. Periostracum with short, fine, bristles aligned in radial rows. Ligament small, triangular, set in shallow resilifer. Hinge teeth robust, 4 anterior and 5 posterior. Inner shell margin smooth. Voucher NHM_453 shell length 4.6 mm, height 4.3mm (Fig. 15).

######### Genetic data.

GenBank NHM_453 18S-MF157498, COI-MF157524.

######### Remarks.

Dissimilar in shape and periostracal bristle configuration to any recorded Eastern Pacific deep-water species (Coan & Valentich-Scott 2012). However, shape and number of hinge teeth are known to change with age/size in *Limopsis* species. In molecular analysis (Fig. 17) forms part of a well supported monophyletic clade with other *Limopsis* species and aligns closest to *Limopsis marionensis* Smith, 1885 from depths of 40–1000 m in the Southern Ocean. No genetic matches on GenBank.

######### Ecology.

Found in polymetallic nodule province.

##### Pectinoida

###### Propeamussiidae Abbott, 1954

####### *Catillopecten* Iredale, 1939

######## Catillopecten

Taxon classificationAnimaliaPholadomyoidaPropeamussiidae

sp. (NHM_105)

######### Material examined.

NHM_105 NHMUK 20170070, collected 2013-10-11, 13.79335 -116.70308, 4081 m. http://data.nhm.ac.uk/object/24f5c5bb-e419-48ef-baaa-4a6493f691d9

######### Description.

**Figure 16.**
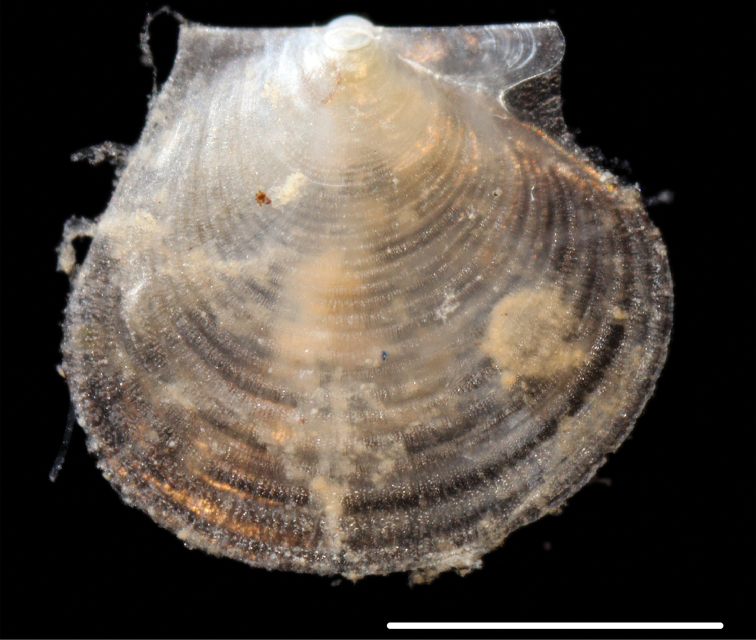
*Catillopecten* sp. (NHM_105) live after recovery. Scale bar: 1 mm. Image attribution Glover, Dahlgren & Wiklund, 2017.

Small, thin-shelled, subcircular. Right valve flat, left valve slightly convex. Both valves with commarginal undulations that become stronger towards the margin, fine radial striations on both valves. Well defined anterior auricle and byssal notch. Voucher NHM_105 1.8 mm shell length, height 1.5 mm (Fig. 16).

######### Genetic data.

GenBank NHM_105 18S-MF157469.

######### Remarks.

**Figure 17.**
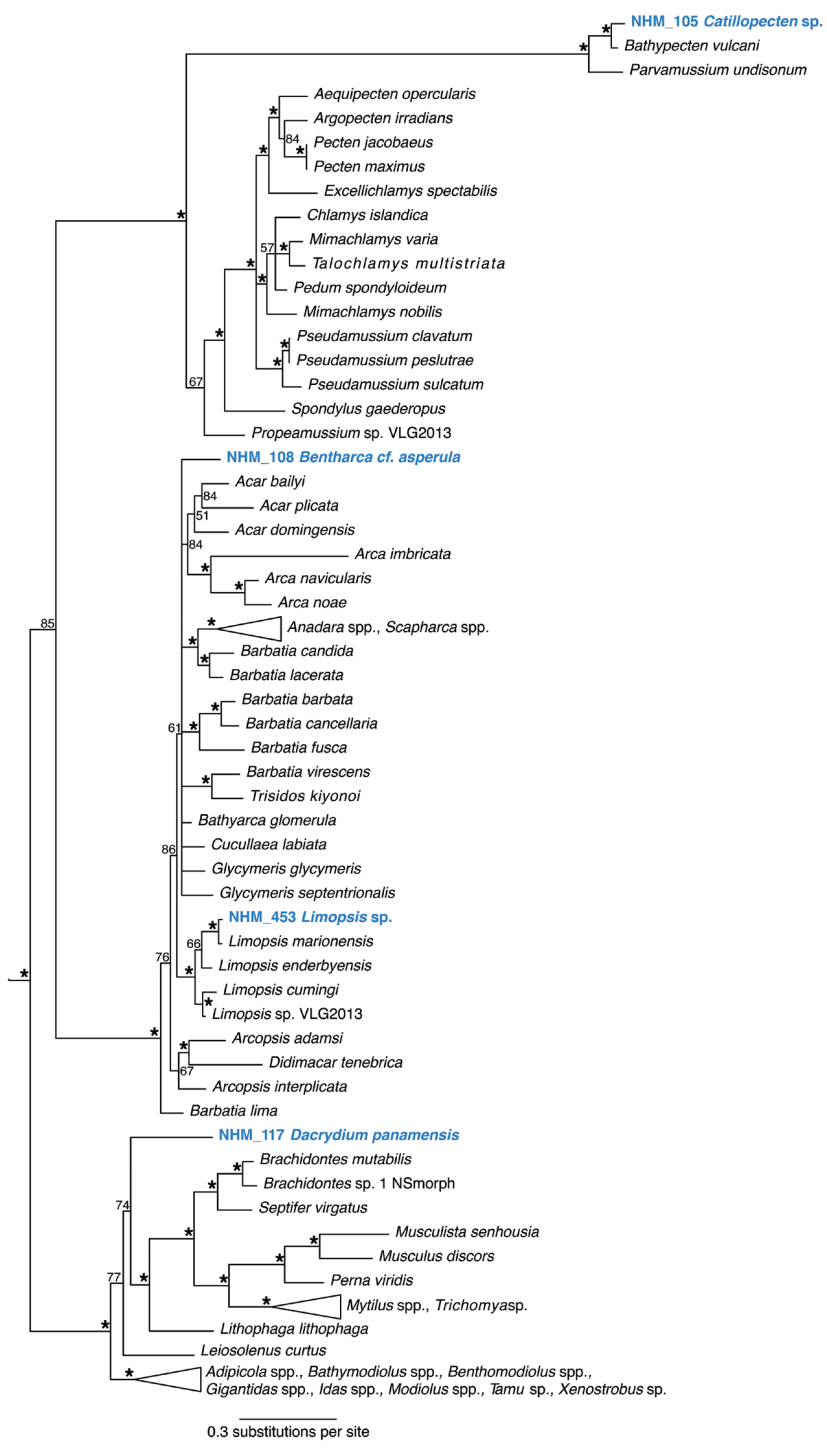
Phylogenetic analysis of Bivalvia: Pteriomorpha. 50% majority rule consensus tree from the Bayesian analyses using 18S and COI. Asterisks denotes support values of 95 or above.

Holotype (ZMUC) from Gulf of Panama, 3270–3670 m Galathea stn 726, figured by Coan and Valentich-Scott (2012 pl. 100). In the molecular tree it groups with two other species of Propeamussidae on a long branch and distinct from other Pectinoida, but a GenBank species (VLG_2013) identified as *Propeamussium* sp. is distinct from these (Fig. 17). Henk H. Dijkstra (Naturalis Biodiversity Center in Leiden, Netherlands) advised on identification of this species. Forms a unique monophyletic clade distinct from all other AB01 specimens. No genetic matches on GenBank.

######### Ecology.

Found in polymetallic nodule province.

##### Caudofoveata

###### Prochaetodermatidae Salvini-Plawen, 1975

####### Prochaetodermatidae

Taxon classificationAnimaliaPholadomyoidaProchaetodermatidae

sp. (NHM_344)

######## Material examined.

NHM_344 NHMUK 20170071.1-2, collected 2013-10-17, 13.75583 -116.48667, 4076 m. http://data.nhm.ac.uk/object/e68608f9-4b83-4eb9-89f2-0de4f89c21b0

######## Description.

**Figure 18.**
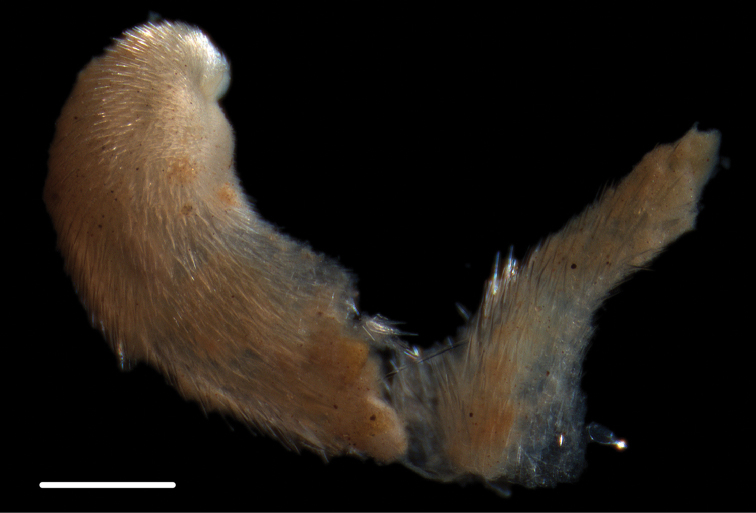
Prochaetodermatidae sp. (NHM_344), abyssal aplacophoran mollusc, imaged after preservation. Scale bar: 0.5 mm. Image attribution Glover, Dahlgren & Wiklund, 2017.

Voucher NHM_344 (Fig. 18) partially broken aplacophoran mollusc, maximum width 0.8 mm, length of fragment ~2.5 mm. Posterior body end lacking. Anterior body intact, with indistinct neck region. Trunk partly damaged. Trunk sclerites are scales with a slender tip confluent with the broad blade without a distinct shoulder region. Tip with keel, triangular in cross section. Blade without sculpture. Data and material, including a permanent preparation of sclerites (1 slide), made available for future study.

######## Genetic data.

GenBank NHM_344 16S-MF157462.

######## Remarks.

The specimen has the typical body shape and sclerite type of Prochaetodermatidae.

######## Ecology.

Found in polymetallic nodule province. Burrows in soft sediment.

#### Monoplacophora

##### Neopilinidae Knight & Yochelson, 1958

###### *Veleropilina* Starobogatov & Moskalev, 1987

####### Veleropilina oligotropha

Taxon classificationAnimaliaPholadomyoidaNeopilinidae

(Rokop, 1972)

######## Material examined.

NHM_405 NHMUK 20170072, collected 2013-10-20, 13.86328 -116.54885, 4050 m. http://data.nhm.ac.uk/object/bf968b01-1991-43b7-87e4-25da4d5a9dc5

######## Description.

**Figure 19.**
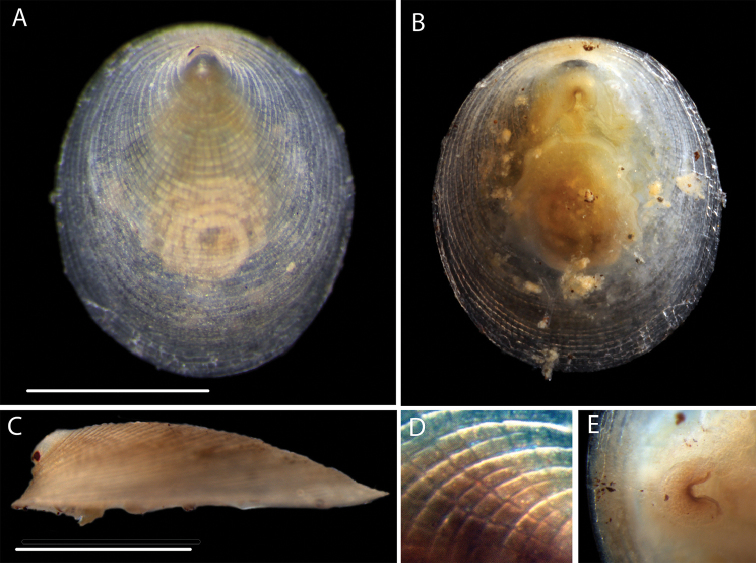
*Veleropilina oligotropha* (Rokop, 1972) Specimen NHM_405. **A** Dorsal view of living specimen **B** Ventral view of living specimen. **C** Lateral view of ethanol-preserved specimen **D** Dorsal shell sculpture detail, just below apex **E** Ventral view of mouth and shell margin. Scale bars: 1mm. Image attribution Glover, Dahlgren & Wiklund, 2017.

Shell transparent, sculpture is reticulate, reticulation not covering the smooth apical area. Voucher specimen NHM_405, specimen length 2.2 mm, specimen width 1 mm (Fig. 19).

######## Genetic data.

GenBank NHM_405 16S-MF157465, 18S-MF157495, COI-MF157522.

######## Remarks.

Morphologically agrees with description of *Veleropilina oligotropha* (Rokop, 1972) described from ~6000 m water depth in the central North Pacific.

**Figure 20.**
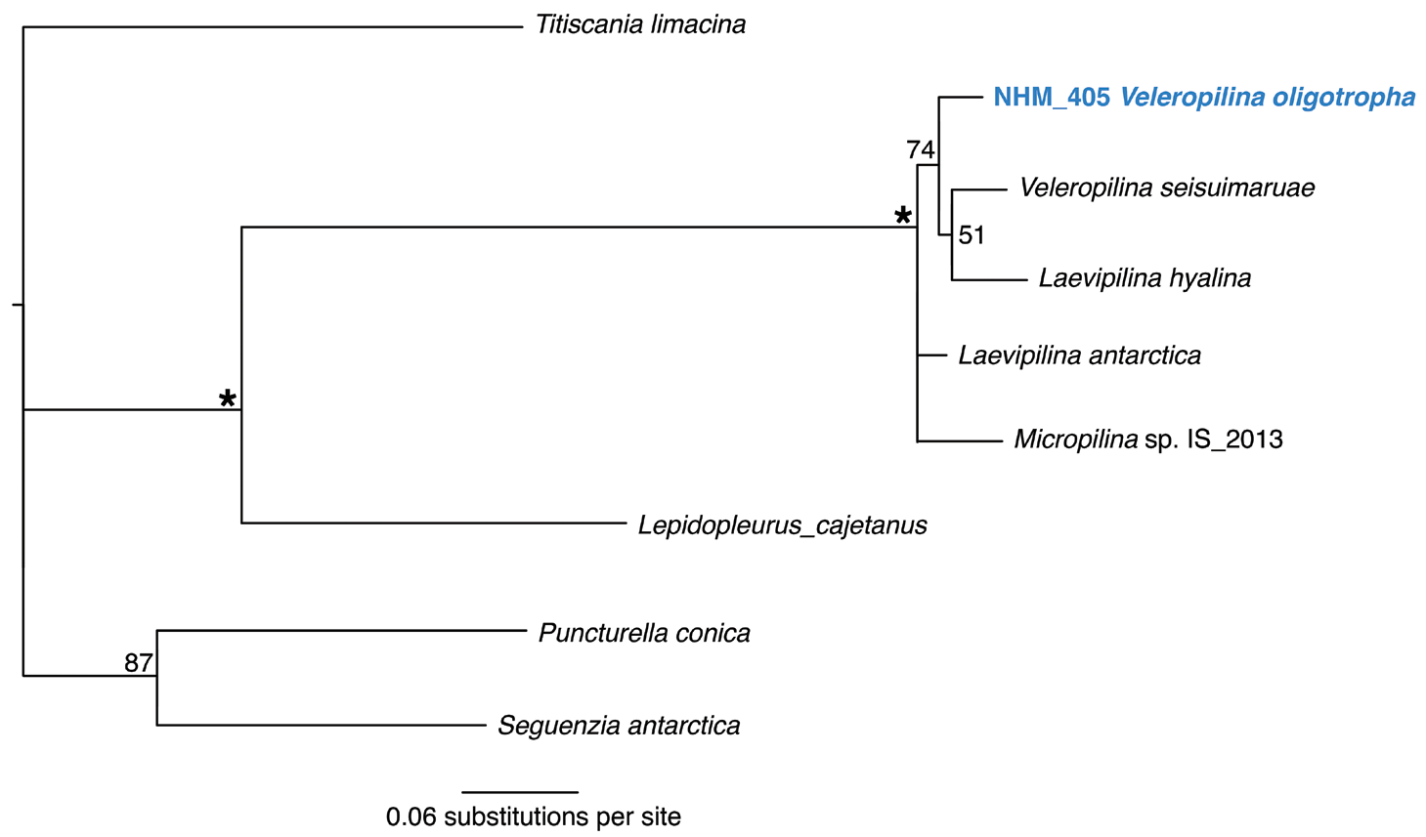
Phylogenetic analysis of Monoplacophora. 50% majority rule consensus tree from the Bayesian analyses using 16S. Asterisks denotes support values of 95 or above.

Forms a unique monophyletic clade distinct from all other AB01 specimens. No genetic matches on GenBank. In the molecular analyses based on the 16S gene, the Monoplacophora clade is strongly supported, but internal branches are unresolved or, when clades are present, they have low support (Fig. 20).

######## Ecology.

Specimen collected from an epibenthic sledge tow across region of sediment and polymetallic nodules. Rokop (1972) did not observe the species directly on nodules, they were just recovered from the epibenthic sledge sample, as was the case in this study. The importance of the nodules as a habitat for the species remains uncertain until they are directly observed live on the seafloor.

#### Polyplacophora

##### Leptochitonidae Dall, 1899

###### *Leptochiton* Gray, 1847

####### Leptochiton macleani

Taxon classificationAnimaliaPholadomyoidaLeptochitonidae

Sirenko, 2015

######## Material examined.

NHM_446 NHMUK 20170073.1-2, collected 2013-10-20, 13.86367 -116.54432, 4050 m. http://data.nhm.ac.uk/object/d69b581d-8a79-4c4d-8f70-88b2ec07d86e

######## Description.

**Figure 21.**
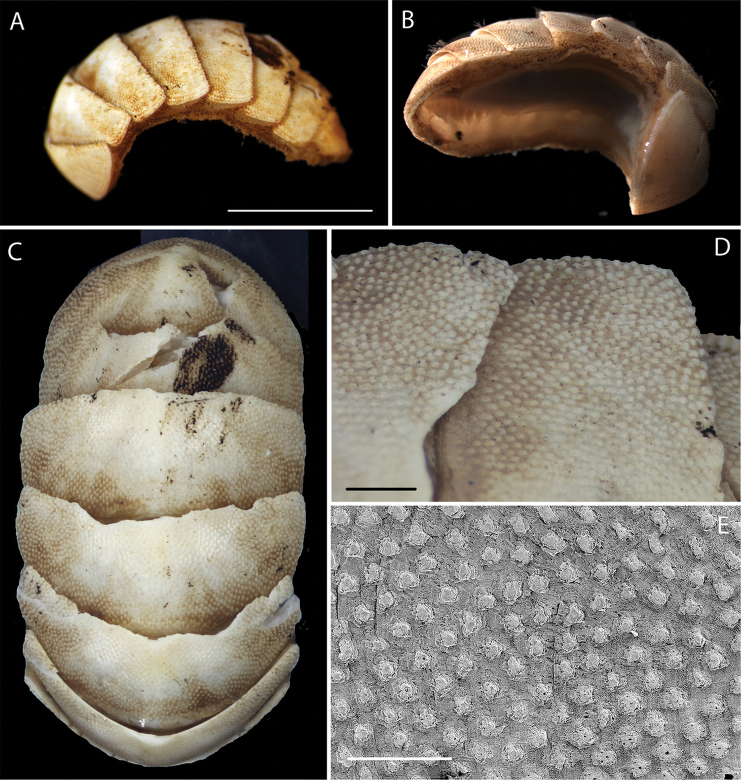
*Leptochiton macleani* Sirenko, 2015. NHM_446 voucher specimen. **A** Live specimen (lateral view) after recovery from the ROV scoop sample **B** Preserved specimen (ventro-lateral view) following DNA extraction **C** Dorsal view **D** surface detail **E** SEM of tegmentum surface and pores. Scale bars: 4 mm (**A**); 0.5 mm (**D**); 0.3 mm (**E**). Image attribution Glover, Taylor, Ikebe, Dahlgren & Wiklund, 2017.

The form and pattern of tegmental granules together with the three aesthete pores are most similar to the images of *Leptochiton macleani* (Sirenko, 2015: figs 34–36). Voucher NHM_446 length approx 10 mm, width 3.2 mm (Fig. 21).

######## Genetic data.

GenBank NHM_446 16S-MF157466, 18S-MF157497, COI-MF157523.

######## Remarks.

**Figure 22.**
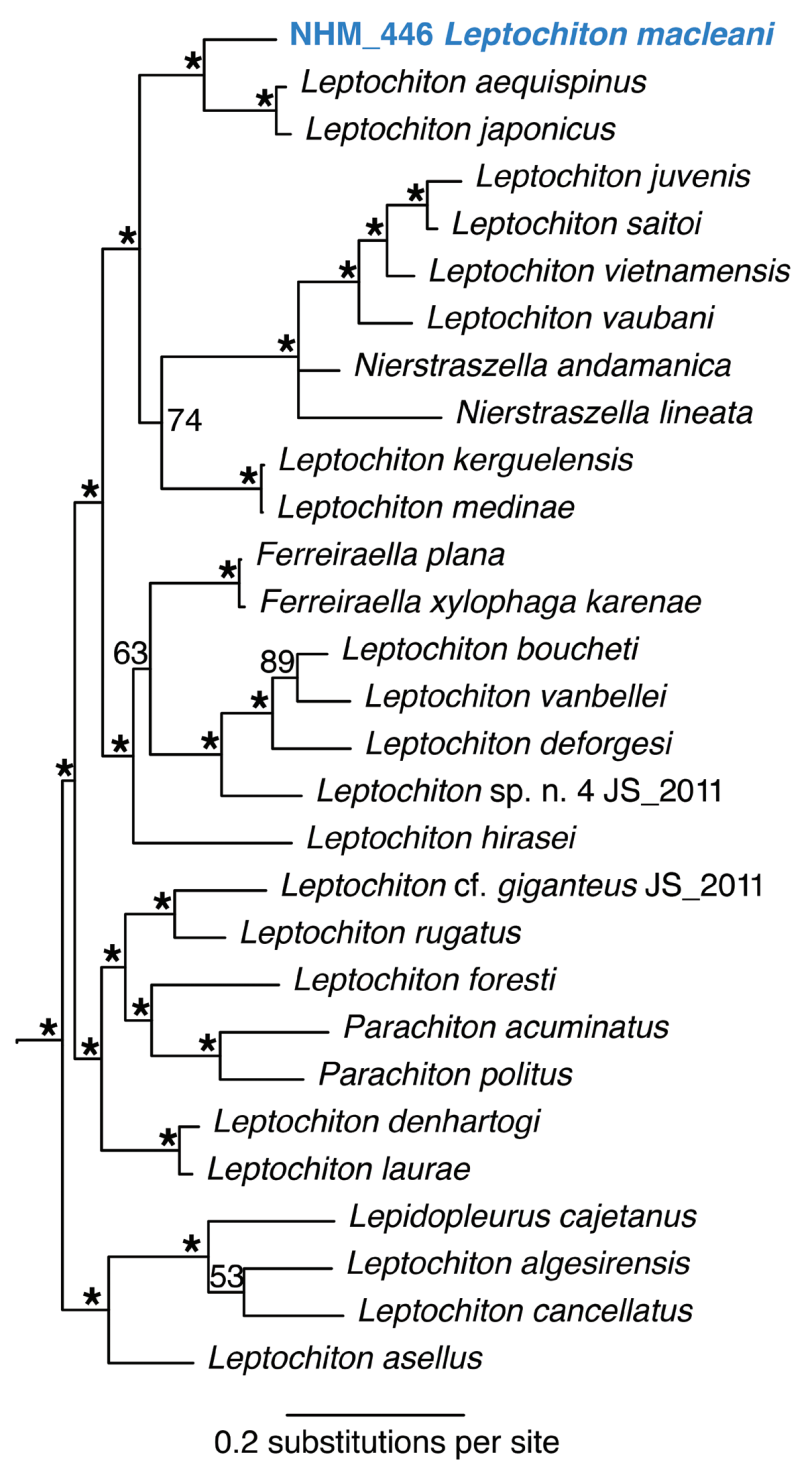
Phylogenetic analysis of Leptochitonidae, Polyplacophora. 50% majority rule consensus tree from the Bayesian analyses, combining 18S and COI. Asterisks denotes posterior probability values of 95 or above.

Sirenko (2015) has recently reviewed *Leptochiton* of the southeastern Pacific Ocean and described several new species that had been previously confounded with *Leptochiton belknapi* Dall, 1878. The specimen morphologically matches *Leptochiton macleani*, type locality Peru-Chile Trench, East Pacific, 4600 m depth. Forms a unique monophyletic clade distinct from other AB01 specimens. No genetic matches on GenBank. In the molecular analyses based on the 18S and COI genes, it falls with strong support as sister taxon to two other *Leptochiton* species, but in the phylogenetic tree the genus *Leptochiton* is not monophyletic (Fig. 22).

######## Ecology.

Specimen collected from an ROV scoop in region of sediment and polymetallic nodules, presumed living associated or on the nodule surface, but not directly observed doing so.

#### Scaphopoda

##### Dentaliida Starobogatov, 1974

###### Dentaliidae Children, 1834

####### *Fissidentalium* Fischer, 1885

######## Fissidentalium

Taxon classificationAnimaliaPholadomyoidaDentaliidae

sp. (NHM_261)

######### Material examined.

NHM_261 NHMUK 20170074, collected 2013-10-17, 13.75583 -116.48667, 4076 m. http://data.nhm.ac.uk/object/679fa0ca-d647-446d-87c5-e8d33949efe2

######### Description.

**Figure 23.**
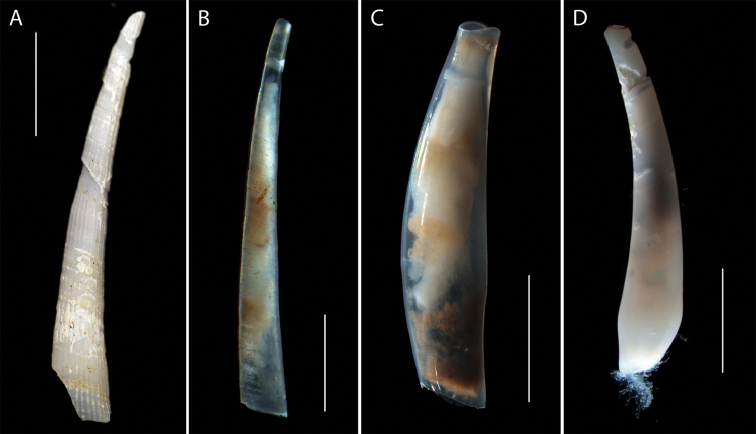
Scaphopoda spp. **A** *Fissidentalium* sp. (NHM_261) live specimen. **B** Gadilida sp. (NHM_192) live specimen **C** *Gadila* sp. (NHM_345) live specimen **D** Gadilida sp. (NHM_132) live specimen. Scale bars: 5 mm (**A, D**); 1 mm (**B**); 2 mm (**C**). Image attribution Glover, Dahlgren & Wiklund, 2017.

A damaged shell with rib features and curvature similar to *Fissidentalium* species (see Scarabino, 1995). Voucher NHM_261, poor preservation, length 21 mm, maximum width 3.1 mm (Fig. 23A).

######### Genetic data.

GenBank NHM_261 16S-MF157461, 18S-MF157489, COI-MF157511.

######### Remarks.

**Figure 24.**
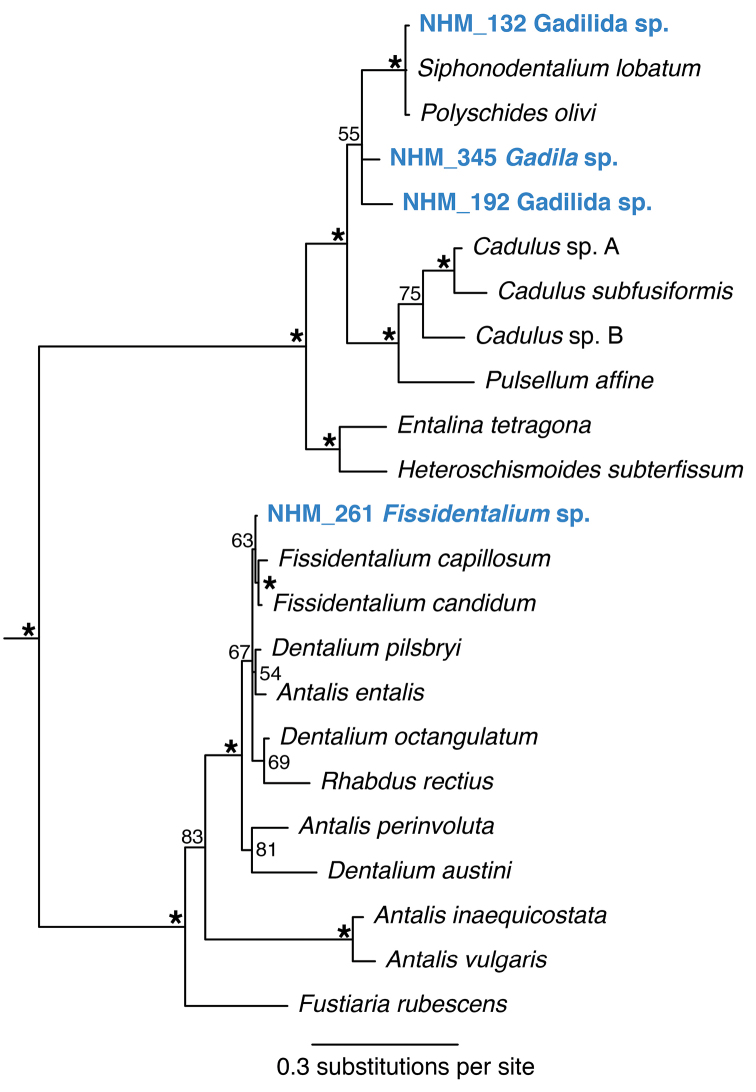
Phylogenetic analysis of Scaphopoda. 50% majority rule consensus tree from the Bayesian analyses using 18S. Asterisks denotes support values of 95 or above.

Forms a unique monophyletic clade distinct from other AB01 specimens. In the molecular analysis it groups with other *Fissidentalium* species, but with very low support. No genetic matches on GenBank. Phylogenetic tree supports placement in order Dentaliida, family Dentaliidae (Fig. 24). Genetic data and imagery provided to facilitate future study.

######### Ecology.

Specimen collected from an epibenthic sledge tow across region of sediment and polymetallic nodules.

##### Gadilida Starobogatov, 1974

###### Gadilida

Taxon classificationAnimaliaPholadomyoidaDentaliidae

sp. (NHM_192)

####### Material examined.

NHM_192 NHMUK 20170075, collected 2013-10-13, 13.93482 -116.55018, 4082 m. http://data.nhm.ac.uk/object/fc0e3ae8-9cce-46a0-bb8b-fafe0e2cb46b

####### Description.

Slender, smooth, transparent, annular growth increments, maximum diameter at mouth. Voucher NHM_192, length 4 mm, maximum width 0.5 mm (Fig. 23B).

####### Genetic data.

GenBank NHM_192 16S-MF157459, 18S-MF157483.

####### Remarks.

Forms a unique monophyletic clade distinct from other AB01 specimens. No genetic matches on GenBank. Phylogenetic tree (Fig. 24) supports placement in order Gadilida with NHM_345. Genetic and image data made available for future study when better specimens available.

####### Ecology.

Specimen collected from an epibenthic sledge tow across region of sediment and polymetallic nodules.

#### Gadilidae Stoliczka, 1868

##### *Gadila* Gray, 1847

###### Gadila

Taxon classificationAnimaliaPholadomyoidaGadilidae

sp. (NHM_345)

####### Material examined.

NHM_345 NHMUK 20170076, collected 2013-10-17, 13.75583 -116.48667, 4076 m. http://data.nhm.ac.uk/object/c301a72f-54cb-435e-8aae-17cf4d37675f

####### Description.

Short, glossy, transparent, maximum diameter near centre, ventral side curved, dorsal side near straight. Mouth simple, oblique. NHM_345 voucher specimen length 6 mm, width 1.4 mm (Fig. 23C).

####### Genetic data.

GenBank NHM_345 16S-MF157463, 18S-MF157493, COI-MF157518.

####### Remarks.

Forms a unique monophyletic clade distinct from other AB01 specimens. No genetic matches on GenBank. Phylogenetic tree supports placement in order Gadilida (Figure 24). Genetic and image data made available for future study when better specimens available.

####### Ecology.

Specimen collected from an epibenthic sledge tow across region of sediment and polymetallic nodules.

###### Gadilida

Taxon classificationAnimaliaPholadomyoidaGadilidae

sp. (NHM_132)

####### Material examined.

NHM_132 NHMUK 20170077, collected 2013-10-11, 13.75833 -116.69852, 4080 m. http://data.nhm.ac.uk/object/6a1906d9-9ed1-4f6e-a0cf-2d53e2289a01

####### Description.

Shell slender, smooth, fairly transparent, increasing in diameter to a maximum about 2.5 mm from the anterior aperture, then decreasing towards the mouth. NHM_132 voucher specimen length 16.6 mm, max width 3 mm (Fig. 23D).

####### Genetic data.

GenBank NHM_132 16S-MF157456, 18S-MF157472.

####### Remarks.

Forms a unique monophyletic clade distinct from other AB01 specimens. No genetic matches on GenBank. Phylogenetic tree (Fig. 24) supports placement in order Gadilida. Genetic and image data made available for future study when better specimens available.

####### Ecology.

Specimen collected from an epibenthic sledge tow across region of sediment and polymetallic nodules.

#### Solenogastres

##### Acanthomeniidae Salvini-Plawen, 1978

###### Acanthomeniidae

Taxon classificationAnimaliaPholadomyoidaAcanthomeniidae

sp. (NHM_367)

####### Material examined.

NHM_367 NHMUK 20170078.1-2, collected 2013-10-19, 13.93307 -116.71628, 4182 m. http://data.nhm.ac.uk/object/c0577fc9-7302-4fec-bc8c-87a17a38bc91

####### Description.

**Figure 25.**
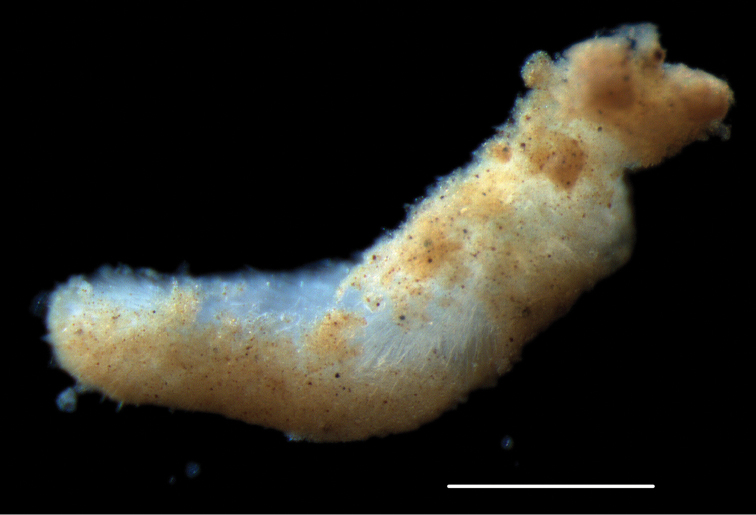
Acanthomeniidae sp. (NHM_367). Living aplacophoran-like mollusc specimen recovered from sledge sample. Scale bar: 0.5 mm. Image attribution Glover, Dahlgren & Wiklund, 2017.

Voucher specimen NHM_367, small solenogaster specimen, anterior end lacking; fragment ca. 2.5 mm long and 0.5 mm in maximum diameter (Fig. 25). Main epidermal sclerites are slender, elongate and pointed scales with a thin, symmetrical rim, and hollow acicular spicules with voluminous cavities, thin walls, and short, pointed tips. Data and material, including a permanent preparation of sclerites (1 slide), made available for future study.

####### Genetic data.

GenBank NHM_367 COI-MF157519.

####### Remarks.

The combination of scales and hollow spicules as main epidermal sclerites is diagnostic for the family Acanthomeniidae. Forms a unique monophyletic clade distinct from other AB01 specimens (Fig. 27). No genetic matches on GenBank.

####### Ecology.

Specimen collected from an epibenthic sledge tow across region of sediment and polymetallic nodules.

#### Pruvotinidae Heath, 1911

##### Lophomeniinae Salvini-Plawen, 1978

###### Lophomeniinae

Taxon classificationAnimaliaPholadomyoidaPruvotinidae

sp. (NHM_027)

####### Material examined.

NHM_027 NHMUK 20170079.1-2, collected 2013-10-09, 13.8372 -116.55843, 4336 m. http://data.nhm.ac.uk/object/319fd186-b07f-4be7-986c-b96c20f63723

####### Description.

**Figure 26.**
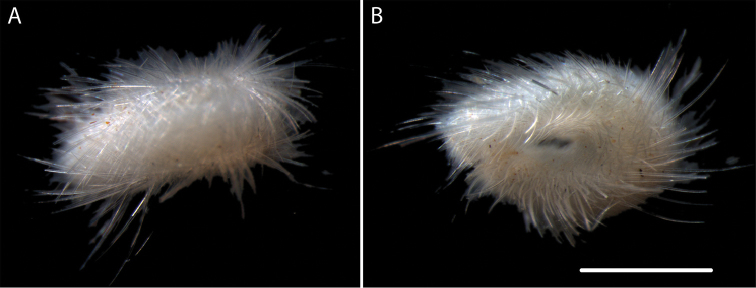
Lophomeniinae sp. (NHM_027) **A** Dorsal view of preserved specimen **B** Preserved specimen (ventro-lateral view) following DNA extraction. Scale bar: 0.5 mm. Image attribution Glover, Dahlgren & Wiklund, 2017.

Voucher specimen NHM_027, small, probably juvenile, solenogaster specimen (Fig. 26). Main epidermal sclerites are very long hollow acicular spicules with simple pointed tips. Spicules slender, s-shaped and thin-walled; tips long and thin. Leaf-shaped pedal scales present. Data and material, including a permanent preparation of sclerites (1 slide), made available for future study.

####### Genetic data.

GenBank NHM_027 COI-MF157500.

####### Remarks.

**Figure 27.**
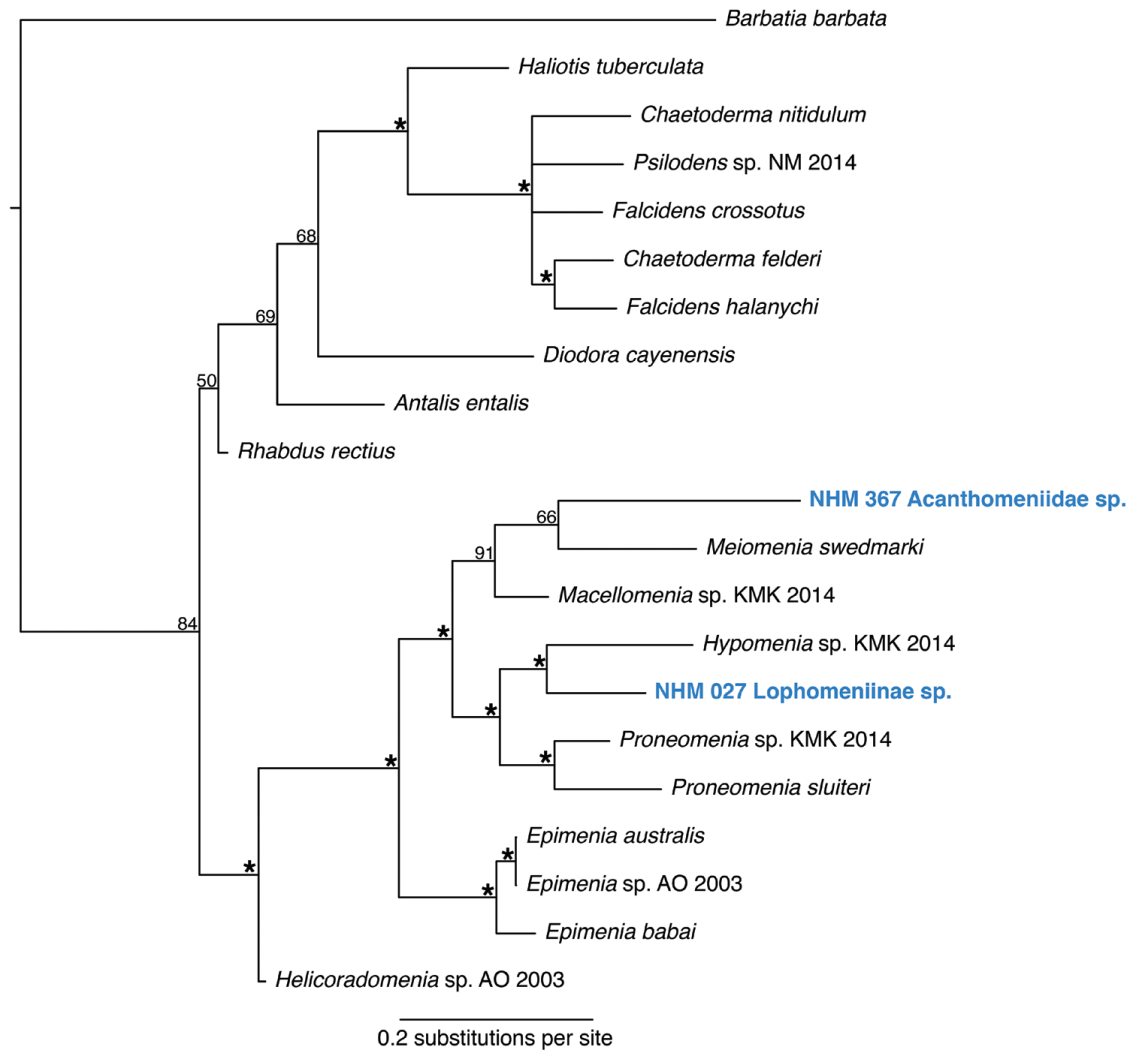
Phylogenetic analysis of Solenogastres, 50% majority rule consensus tree from the Bayesian analyses using COI. Asterisks denotes support values of 95 or above.

Forms a unique monophyletic clade distinct from other AB01 specimens (Fig. 27). No genetic matches on GenBank. Body shape and sclerites are characteristic for the family Pruvotinidae and indicative of the subfamily Lophomeniinae. Placement as sister to *Hypomenia*, another pruvotinid species, in the phylogenetic analysis (Fig. 27) confirms the family-level affiliation.

####### Ecology.

Specimen collected from an epibenthic sledge tow across region of sediment and polymetallic nodules.

## Discussion

Only one record of benthic mollusc taxa in the CCZ is hitherto reported on OBIS (OBIS 1017; iobis.org), with a further four just south of CCZ. In this study we report 42 records for 21 taxa, of which one is described as a new species. All our data and material from this study are made publicly available through this publication, and through depositing DNA extractions and tissue for further molecular analyses in the Molecular Collections Facility as well as morphological vouchers at the Natural History Museum in London, UK.

Mollusca is a diverse group with its members having very differing life histories, and in this study there are representatives of both sediment-dwelling species and nodule fauna. Not much is known about the mollusc species distribution and connectivity within the CCZ, an information deficit that makes it impossible to assess impact from anthropogenic activities. Genetic data is crucial for distribution analyses as some taxa look very similar and can be difficult to separate to species level based on morphology only, e.g. the new species *Ledella knudseni* and its sister taxon *Ledella* sp. (NHM_381). In our study we have used a precautionary approach when reporting taxa that are preliminary identified as described species with type locality far from CCZ, e.g. our Bentharca cf. asperula which is very similar to *Bentharca asperula* with type locality in Gulf of Mexico. Without genetic information from specimens collected at the type locality, we can not rule out that ours is a different species despite the similarity in morphology.

The protobranch bivalve *Nucula profundorum* is the most abundant bivalve mollusc in our samples, and population connectivity analyses are underway (Dahlgren et al. in prep). Morphologically it is identical to type material of the original *Nucula profundorum*, which was described from collections of HMS Challenger in the mid-North Pacific (36°N, 178°E) at about 3750 m depth (Fig. 10), and although our specimens were collected further south and east, the depth is almost the same. However, as we compare our sequences with published *N. profundorum* sequences on GenBank it is obvious that those two are different species. The sequences already published on GenBank come from specimens collected at about 1000 m depth off San Diego. Based on morphological similarity only, and the general observation that depth is a stronger barrier to dispersal than geographic distance (e.g Etter & Rex, 1990), our hypothesis is that our specimens are likely to correspond to *N. profundorum* and that the sequences attributed to *N. profundorum* on GenBank are erroneously identified.

There are very few DNA sequences from a few faunal groups from the CCZ available on GenBank, e.g. echinoderms (Glover et al. 2016b), cnidarians (Dahlgren et al. 2016) and polychaetes and crustaceans (Janssen et al. 2015). With our study including both morphological and molecular data we add greatly to our knowledge of genetic information in the CCZ and aim to improve the taxonomic understanding of benthic fauna in the CCZ to get a better picture of the distribution of taxa. These are essential data for the establishment of conservation strategies in the light of future mineral extraction.

## Supplementary Material

XML Treatment for Myonera

XML Treatment for Thyasira

XML Treatment for Vesicomya galatheae

XML Treatment for Bathyspinula calcar

XML Treatment for Ledella knudseni

XML Treatment for Ledella

XML Treatment for Nucula profundorum

XML Treatment for Yoldiella

XML Treatment for Bentharca cf. asperula

XML Treatment for Dacrydium panamensis

XML Treatment for Limopsis

XML Treatment for Catillopecten

XML Treatment for Prochaetodermatidae

XML Treatment for Veleropilina oligotropha

XML Treatment for Leptochiton macleani

XML Treatment for Fissidentalium

XML Treatment for Gadilida

XML Treatment for Gadila

XML Treatment for Gadilida

XML Treatment for Acanthomeniidae

XML Treatment for Lophomeniinae
